# Additional Valine and Isoleucine Impact Growth Performance, Intestinal Health, and Muscle Growth in Broilers Under Necrotic Enteritis Challenges

**DOI:** 10.3390/ani15182641

**Published:** 2025-09-09

**Authors:** Doyun Goo, Woo Kyun Kim

**Affiliations:** Department of Poultry Science, University of Georgia, Athens, GA 30602, USA; dgoo@uga.edu

**Keywords:** branched-chain amino acids, breast muscle, gut health, microbiome, necrotic enteritis

## Abstract

The current study aims to investigate the effects of additional valine and isoleucine in different necrotic enteritis (NE) challenge intensities. A total of 648 seven-day-old male Cobb 500 were allotted to nine treatments with six replicates. Two different NE intensity experiments (Exp-1 and Exp-2) were conducted. The five treatments for each NE experiment were as follows: (1) non-challenged shared control (NC), (2) NE challenge (NE), (3) 130% additional valine (VAL), (4) 130% additional isoleucine (ILE), and (5) 130% valine and isoleucine (MIX). In both experiments, all NE-challenged groups had decreased body weight gain compared to the NC group (*p* < 0.05). The ILE group in Exp-1 had further reduced body weight compared to the NE group on day 21 (*p* < 0.05). No significant differences were observed in intestinal permeability, jejunal NE lesion score, jejunal *C. perfringens* colony counts, jejunal morphology, jejunal gene expression levels, breast muscle yield, and body mineral compositions among all NE-challenged groups (*p* > 0.05). In conclusion, 130% additional valine and isoleucine hardly mitigated the negative effects of NE. Additional isoleucine may further worsen chicken growth performance, but this effect may vary depending on the intensity of the NE challenge.

## Simple Summary

Necrotic enteritis caused by *Clostridium perfringens* is one of the key factors that negatively affect chicken growth. Branched-chain amino acids play an important role in muscle growth and energy production in broilers, and additional valine and isoleucine can potentially mitigate the negative effects of necrotic enteritis. Therefore, this experiment investigated whether additional valine and isoleucine alleviated the negative effects caused by a necrotic enteritic-challenged condition. Necrotic enteritis infection worsened overall gut health and triggered inflammation, which impaired chicken growth performance. Additional valine and isoleucine did not improve performance but were affected by the intensity of necrotic enteritis infection. However, additional valine could mitigate the loss of bone mineral content due to necrotic enteritis. Regardless of the intensity of necrotic enteritis infection, additional valine and isoleucine were found to have little effect in terms of improving gut health, but changes in bone mineral content require further study.

## 1. Introduction

Necrotic enteritis (NE), caused by *Clostridium perfringens*, is an enteric bacterial disease that causes detrimental effects on poultry growth performance [1]. Particularly, the subclinical form of NE causes enormous economic losses to the poultry industry because it decreases weight gain and feed efficiency in chickens without acute death and apparent symptoms [2,3]. *C. perfringens* is distributed over a wide range of environments and is also present in the small intestine of healthy chickens [4]. The development of NE is caused by toxins secreted by *C. perfringens*, among which the *C. perfringens* type G strain secreting NE B-like toxin (NetB) is known to be the most important virulence factor [5,6,7]. In addition, the appropriate predisposing factors must be present for *C. perfringens* to successfully cause NE in chickens [8]. *Eimeria*, which directly damages intestinal epithelial cells, can have the greatest impact [9,10,11], and other predisposing factors such as stress, farm management, environmental conditions, immunosuppression, diet, and species can also contribute to NE development [12,13,14,15,16,17,18]. Numerous predisposing factors can trigger NE in chickens, but from a different perspective, there are also many candidates that can potentially mitigate NE development, one of which could be a modification in diet AA composition.

Nutritional approaches to prevent NE may be effective because *C. perfringens* also relies on AAs for its growth in the intestinal environment, and methods such as reducing dietary crude protein (CP) levels and regulating dietary amino acid (AA) ratios can be used [19,20]. High CP levels in the diet may be helpful if protein requirements are increased due to intestinal infections, but caution should be taken as *C. perfringens* can also use indigestible protein sources for its proliferation [21]. Xue et al. (2017) reported that growth performance and intestinal NE lesion scores increased together as a result of additional glycine in the diet [22]. On the other hand, when glutamine was added to the diet, most of the negative effects of NE infection were alleviated [23]. In addition, Keerqin et al. (2017) reported that indispensable AAs and CP levels increased by 110% in the diet, which could mitigate the negative effects of NE [24]. This may be because indispensable AAs consequently play an important role in the development of the immune system in chickens [25]. However, despite these plausible effects of AAs, few studies have been conducted assessing the effects of additional dietary branched-chain AAs (BCAAs) and NE challenge in broilers.

Among BCAAs, valine and isoleucine are indispensable AAs in poultry and play important roles in overall chicken growth by promoting muscle synthesis and energy production [26,27]. Despite the important role of BCAAs, these AAs cannot be synthesized in animals and must be supplemented through diet [28]. Particularly in corn/soybean meal-based diets, valine is proposed as the fourth limiting AA, following methionine, lysine, and threonine [29]. Branched-chain AAs play a major role in the skeletal muscle and the liver after absorption due to their unique catabolic mechanism [30,31], but some studies have also investigated their functional roles, such as intestinal health, immunity, and microbiome in chickens [32,33,34,35,36]. However, the functional roles of valine and isoleucine in conditions such as excessive immune response, reduced feed consumption, and deterioration of intestinal health under NE challenge may differ, and their relevance needs to be identified. The hypothesis of the current study was that additional valine and isoleucine in the diet can mitigate the negative effects of NE infection. Therefore, the objective of the current study was to investigate the effects of additional valine and isoleucine under two different NE-challenged conditions on growth performance, breast muscle and lean weight, body mineral composition, intestinal permeability, jejunal lesion score and morphology, jejunal and breast muscle gene expression, and cecal microbiome in broiler chickens.

## 2. Materials and Methods

### 2.1. Chickens, Experimental Design, and Necrotic Enteritis Challenge Model

The study was conducted at the University of Georgia Poultry Research Center (PRC, Athens, GA, USA) and was reviewed and received approval from the Institutional Animal Care and Use Committee (A2021 12-012). On day (d) 7, a total of 648 male Cobb 500 broilers (average body weight = 159 ± 0.2 g) were randomly assigned to nine treatments with six replicates and 12 chickens per cage. On d 14, all chickens (average body weight = 453 ± 2.2 g) were weighed again before NE challenges were applied. In the current study, based on the *E. maxima* + *C. perfringens* NE coinfection challenge model, we separated this model into two different experiments: (1) normal NE challenge (Exp-1); 1 mL of sporulated *E. maxima* 12,500 oocysts (USDA-ARS strain 41A) was orally challenged on d 14 and followed by 1 mL of NetB^+^ 1 × 10^9^ *C. perfringens* (USDA-ARS strain Del-1), administered to the chickens 4 days post inoculation (dpi); and (2) severe NE challenge (Exp-2); the same *E. maxima* challenge method was used as Exp-1, but NetB^+^ 1 × 10^9^ *C. perfringens* inoculums were administered three times on 4, 5, and 6 dpi. Subclinical NE coinfection challenge methods in the current study were used according to a previously reported method [37].

**Figure 1:**
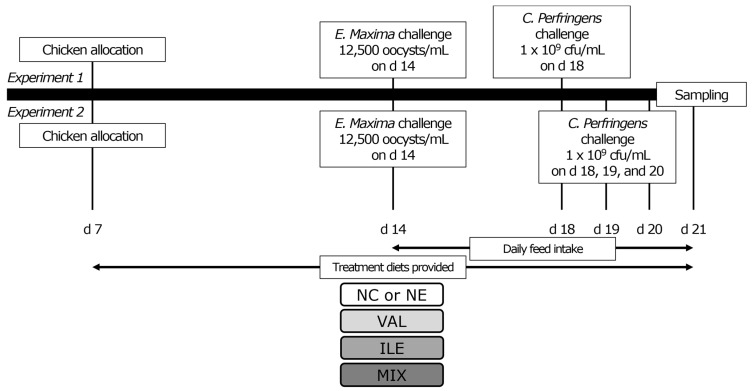
A brief diagram illustrating the progress of the current study. Abbreviations: d, day; NC, non-challenged control (leucine/lysine = 1.12; valine/lysine = 0.76; isoleucine/lysine = 0.65, as a calculated value); NE, NE challenge group; Experiment 1, challenge with 1 mL of 12,500 *E. maxima* oocysts on d 14 followed by 1 mL of *C. perfringens* 1 × 10^9^ challenge on d 18; Experiment 2, challenge with 1 mL of 12,500 *E. maxima* oocysts on d 14 followed by 1 mL of *C. perfringens* 1 × 10^9^ challenge three times on d 18, 19, and 20; VAL, 130% additional valine diet (valine/lysine = 0.98, as a calculated value); ILE, 130% additional isoleucine diet (isoleucine/lysine = 0.84, as a calculated value); MIX, 130% additional valine and isoleucine diet (valine/lysine = 0.98 and isoleucine/lysine = 0.84, as a calculated value).

**Figure 2:**
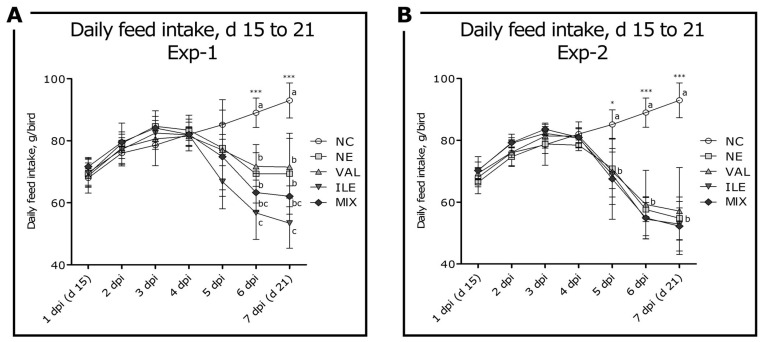
Effects of valine or isoleucine supplementation in two necrotic enteritis challenge experiments on daily feed intake (DFI) trends in broilers from d 15 to 21 (1 to 7 dpi). No significant difference was observed in DFI among the groups before the *E. maxima* challenge on d 14 (*p* > 0.05). ^a–c^ Means in the same dpi column with different superscripts are statistically different (*p*-value < 0.05). Abbreviations: NC, non-challenged control (leucine/lysine = 1.12; valine/lysine = 0.76; isoleucine/lysine = 0.65, as a calculated value); NE, NE challenge group; Exp-1, challenge with 12,500 *E. maxima* oocysts on d 14 followed by *C. perfringens* 1 × 10^9^ challenge on d 18; Exp-2, challenge with 12,500 *E. maxima* oocysts on d 14 followed by *C. perfringens* 1 × 10^9^ challenge three times on d 18, 19, and 20; VAL, 130% additional valine diet (valine/lysine = 0.98, as a calculated value) in NE-challenged conditions; ILE, 130% additional isoleucine diet (isoleucine/lysine = 0.84, as a calculated value) in NE-challenged conditions; MIX, 130% additional valine and isoleucine diet (valine/lysine = 0.98 and isoleucine/lysine = 0.84, as a calculated value) in NE-challenged conditions. (**A**) DFI trend of Exp-1 from 1 to 7 dpi; (**B**) DFI trend of Exp-2 from 1 to 7 dpi. *, *p* < 0.05; ***, *p* < 0.001.

The two experiments consisted of five treatments; both experiments shared the same non-challenged control group. Excluding differences in NE challenge intensities, the composition of the diet was designed equally (Table 1). The five different treatments were as follows: (1) control diet (NC; leucine/lysine ratio = 1.12, valine/lysine ratio = 0.76, and isoleucine/lysine ratio = 0.65), (2) control diet with NE-challenged conditions (NE), (3) 130% additional valine in NE-challenged conditions (VAL; valine/lysine ratio was set to 0.98), (4) 130% additional isoleucine in NE-challenged conditions (ILE; isoleucine/lysine ratio was set to 0.84), and (5) 130% additional valine and isoleucine in NE-challenged conditions (MIX; valine/lysine ratio was set to 0.98 and isoleucine/lysine ratio was set to 0.84). Corn/soybean meal-based mash form experimental diets were formulated and provided ad libitum from d 7 to 21 (Table 1). An analyzed diet composition table, including total AAs and crude protein levels, is shown in Table 2. A brief diagram illustrating the progress of the current study is presented in Figure 1. The chickens were reared in 4-layer nipple-installed battery cages (dimensions: 100 × 36 × 41 cm, length × width × height) and a wide single feeder was used. After weighing on d 14, the stocking density was set at 27.8 chickens/m^2^ by removing 2 chickens per cage. Following the 2021 Cobb Broiler Management Guide [38], temperature and light were automatically controlled until the end of the study on d 21. Body weight (BW) was measured on d 7, 14, and 21, and BW gain (BWG), feed intake (FI), and feed conversion ratio (FCR) were calculated for d 7 to 14, d 15 to 21, and d 7 to 21. Daily feed intake (DFI) was measured every morning and expressed as the DFI trend from d 15 to 21 (1 to 7 dpi) in Figure 2.

### 2.2. Intestinal Permeability and Jejunal Lesion Scores

On d 21 (7 dpi), one chicken per replicate was randomly selected for intestinal permeability, and BW was recorded. Then, 2 mg/mL fluorescein isothiocyanate-dextran (FITC-d; Cat# 46944, Molecular weight 4000; Sigma-Aldrich, Darmstadt, Germany) was orally inoculated following the previously reported methods [39,40]. Prior to sampling, chickens were isolated in separate cages and kept away from feed and water nipples. After 120 min, all chickens were euthanized by cervical dislocation, and blood was obtained directly through cardiac puncture. The blood samples were then completely covered with aluminum foil and stored at room temperature for up to 3 h. Control sera were collected from three non-experimental extra chickens to create a standard curve. Clotted blood sample tubes were centrifuged at 1500× *g* for 12 min, and approximately 1 mL of supernatant was collected. Using the serial dilution method, a standard curve was made ranging from 0 to 2 mg (y=0.0002x−73.95, R^2^ = 0.997). Samples and standard curves (100 μL each) were measured together in a 96 dark flat-bottom well plate at an optical density of 485/525 nm using a microplate reader (Spectra Max 5 microplate reader, Molecular Devices, Sunnyvale, CA, USA) [41]. All serum samples were detected within the standard curve range.

After the growth performance measurement on d 21 (7 dpi), two chickens per cage were randomly selected and euthanized by cervical dislocation for jejunal NE lesion scoring. Jejunal NE lesions were scored on a 0- to 3-point scale following a previously reported procedure [18]. The jejunal segment from the end of the duodenal loop to Meckel’s diverticulum was selected for NE lesion scoring. To prevent bias in the opinion of the investigators, jejunal NE lesion scoring was performed in the order of cage numbers instead of treatment names.

### 2.3. Jejunal C. perfringens Colony Count and Fecal E. maxima Oocyst Count

Jejunal *C. perfringens* colony counting and fecal *E. maxima* oocyst counting were followed by previously reported methods [42,43]. In short, one chicken per cage was randomly selected and euthanized by cervical dislocation, and whole jejunal contents were collected in filter bags (While-Pak, Nasco, Fort Atkinson, WI, USA). Jejunal digesta were collected in quantities of at least 10 mL and up to 20 mL, and one additional chicken was euthanized when jejunal contents were not enough. After that, 10 mL of 0.1% buffered peptone water (BPW; Cat# M614-100G, Himedia Laboratories LLC, Kelton, PA, USA) was added to the filter bag, and the total weight (contents + peptone water + filter bag) was weighed and homogenized for 1 min (Masticator Silver Panoramic, Neutec Group Inc., Farmingdale, NY, USA). The homogenized jejunal content mixture was then transferred into a sterile 96-flat bottom plate and diluted up to 10^−8^ using a serial dilution. One hundred microliters of the diluted intestinal content mixtures in two different dilution wells (NC group: 10^−3^ and 10^−5^; NE-challenged groups: 10^−5^ and 10^−7^) were spread onto Tryptose Sulfite Cycloserine and Shahidi Ferguson Perfringens (TSC/SFP; Cat# M837-500G, Himedia Laboratories LLC, Kelton, PA, USA) agar plates and gently spread for up to 1 min. Subsequently, the plate was turned upside down when the mixture had been spread well on the TSC/SFP agar plates, and then anaerobically incubated at 37 °C for up to 48 h (AnaeroPack™, Thermo Scientific, MA, USA). After the incubation, plates in which the number of *C. perfringens* colonies ranged from 15 to 250 were used for counting.

For the fecal oocyst counting, all feces trays in the battery cages were removed and cleaned 36 h before the feces collection (5 to 6 dpi), and all feces were collected on 7 dpi (d 21). On collection day (d 21), a minimum of 50 g to a maximum of 100 g of mixed total feces were collected in a sample bag. Five grams of mixed feces were then diluted with approximately 35 mL of water in a 50 mL centrifuge tube. Then, 1 mL of the diluted feces sample (feces with tap water) was blended again with 10 mL of a saturated salt solution. Six hundred microliters of the feces mixture were then added to a McMaster oocyst counting chamber (Vetlab Supply, Palmetto Bay, FL, USA). All six columns were used for *E. maxima* oocyst counting [44], and the total *E. maxima* oocyst count was expressed on a log_10_ scale.

### 2.4. Intestinal Villus Height: Crypt Depth Ratio and Goblet Cell Count

Intestinal samples were taken from the same chickens euthanized for the jejunal NE lesion score for the determination of intestinal villus height to crypt depth ratio (VH:CD) and goblet cell counts. Intestinal section sampling and sample preparation methods were performed according to the previously reported method [45]. Briefly, approximately 3 cm of jejunum and ileum sections were washed with phosphate-buffered saline (PBS) and directly fixed in 40 mL of 10% neutral-buffered formalin solution for further analysis. After that, jejunum and ileum sections were embedded in paraffin of about 0.5 cm^3^ size. Fixed intestinal samples were stained with Periodic acid–Schiff (PAS; Cat# 395B, Sigma-Aldrich, Darmstadt, Germany) and counterstained with hematoxylin (Cat# MHS32, Sigma-Aldrich, Darmstadt, Germany), following a previously reported method [46]. To calculate the VH:CD, at least two intact villi and crypts were selected in each image, and their lengths were measured using a BZ microscope (BZ-X810, Keyence, Osaka, Japan). For the intestinal goblet cell counts, the two longest villi were measured and averaged, and incomplete villi were excluded from the measurement.

### 2.5. Breast Muscle Yield and Body Composition Analysis Using DEXA

After the growth performance measurement on d 21 (7 dpi), one average BW chicken per cage was selected and euthanized by cervical dislocation, and breast muscle yield and Dual Energy X-ray Absorptiometry (DEXA, GE Healthcare, Chicago, IL, USA) scanning was performed. For the determination of breast muscle yield, the weight of one euthanized chicken was first measured before harvesting the breast muscle. Both breast muscle weight and relative weight were expressed following a previously reported method [47].

For the DEXA scanning, a calibration step was first performed, and then two-dimensional scanning was conducted. The scanning range was set for small animals (196.6 × 62.0 cm, length × width), and the exposure setup was configured to a voltage of 76 kV, a current of 0.15 mA, and a dose of 1.8 μGy, following a previously reported setup [48]. Up to 15 chickens were placed in one DEXA scanning operation, and all selected chickens were scanned within 60 min to minimize weight loss in dead chickens. After scanning, individual images of chickens were collected by setting regions of interest. Bone mineral density (BMD), bone mineral content (BMC), total tissue weight, lean weight, and fat weight were measured [49]. Bone mineral content ratios (BMCRs) were calculated separately to correct BMC levels due to weight differences [47].

### 2.6. Jejunum and Breast Muscle Gene Expression Using qRT-PCR

In the current study, the expression of AA transporters, inflammatory cytokines, and toll-like receptor (TLR)/nuclear factor kappa B (NFκB) signaling pathway-related, mechanistic target of rapamycin (mTOR) pathway-related, and BCAA catabolism-related genes were evaluated. Jejunal section and breast muscle samples were taken from the same chickens euthanized for the determination of breast muscle yield on d 21 (7 dpi) for quantitative real-time reverse transcriptase polymerase chain reaction (qRT-PCR) analysis. Each of the jejunum and breast muscle samples were rinsed with sterile PBS, placed immediately in liquid nitrogen, and stored at −80 °C for further analysis. The RNA extraction method followed a previously reported method [50]. For sample preparation, approximately 100 mg of jejunum and breast muscle samples were cut and placed in 1.7 mL screw cap tubes, and 1 mL of QIAzol Lysis reagent (Cat# 79306, Qiagen, Valencia, CA, USA) and approximately 20–30 1.0 mm diameter zirconia/silica beads (BioSpec Products, Bartlesville, OK, USA) were directly added to the tubes. Subsequently, the sample mixture was homogenized for a maximum of 120 s at 2400× *g* using a bead beater (Biospec Products, Bartlesville, OK, USA). Then, RNA was extracted from prepared samples according to the manufacturer’s protocol. After the RNA extraction, the RNA pellet was diluted in 380 μL (for jejunum) and 180 μL (for breast muscle) of HyPure™ Molecular Biology Grade Water (Cat# SH30538.02, Cytiva, HyClone Laboratories, South Logan, UT, USA) to make a final RNA concentration range between 500 and 1000 ng/μL. The quantity and purity of the diluted RNA were then checked using a Nanodrop spectrophotometer (Thermo Scientific™ NanoDrop™ Eight Spectrophotometer, Thermo Scientific, Waltham, MA, USA). On the same day, cDNA was synthesized from the RNA using a cDNA Reverse Transcription kit (Cat# 4368813, Applied Biosystems, Foster City, CA, USA). The PCR cycle for the cDNA synthesis procedure was as follows: 10 min at 25 °C, then 2 h at 37 °C, followed by 85 °C for 5 min, and the total volume of the mixture was 20 μL. After all PCR steps were performed, the final synthesized cDNA was diluted in 180 μL of Molecular Biology Grade Water and stored at 4 °C for up to 2 weeks. For the qRT-PCR analyses, the QuantStudio™ 3 Real-Time PCR (Applied Biosystems, Foster City, CA, USA) and the iTaq™ Universal SYBR^®^ Green Supermix (SYBR; Cat# 1725125, Bio-Rad Laboratories Inc., Waltham, MA, USA) were used. The final mixture volume was set to 10 μL, consisting of 5 μL of SYBR, 2.5 μL of cDNA, 0.25 μL of forward primer, 0.25 μL of reverse primer, and 2 μL of Molecular Biology Grade Water. The thermal cycle settings for all qRT-PCR analyses followed a previously reported method [48]. Two reference genes, *beta-actin* and *glyceraldehyde-3-phosphate dehydrogenase* (*GAPDH*), were used for data calculation, and data were expressed as relative fold changes compared to the NC group. The sequence information is presented in Table S1, and product sizes of all primers in the current study were designed within 100 to 150 base pairs.

### 2.7. Cecal Microbiome Analysis

Cecal contents were taken from the same chickens euthanized for breast muscle yield and gene expression analysis on d 21 (7 dpi) for cecal microbiome analysis. The cecal content was collected and then directly placed in liquid nitrogen and stored at −80 °C for further analysis. Five grams of cecal content were used for DNA extraction, following a previously reported procedure [51]. On extraction day, a QIAamp^®^ DNA Stool Mini Kit (Cat# 51604, Qiagen GmbH, Hilden, Germany) was used for cecal DNA extraction, according to the manufacturer’s protocol. The concentration of the cecal DNA was then checked using a Thermo Scientific™ NanoDrop™ Eight Spectrophotometer before analysis. DNA samples were analyzed at LC Science (Houston, TX, USA) to perform 16S rRNA gene sequencing [52]. Subsequently, the 16S rRNA gene sequences were processed and analyzed using Quantitative Insights into Microbial Ecology 2 version 2022.02, following the methods of previously reported studies [53,54]. After data processing, RStudio software (R Version 4.2.2, RStudio PBC, Boston, MA, USA) was used for visualizing data.

### 2.8. Statistical Analysis

**Figure 3:**
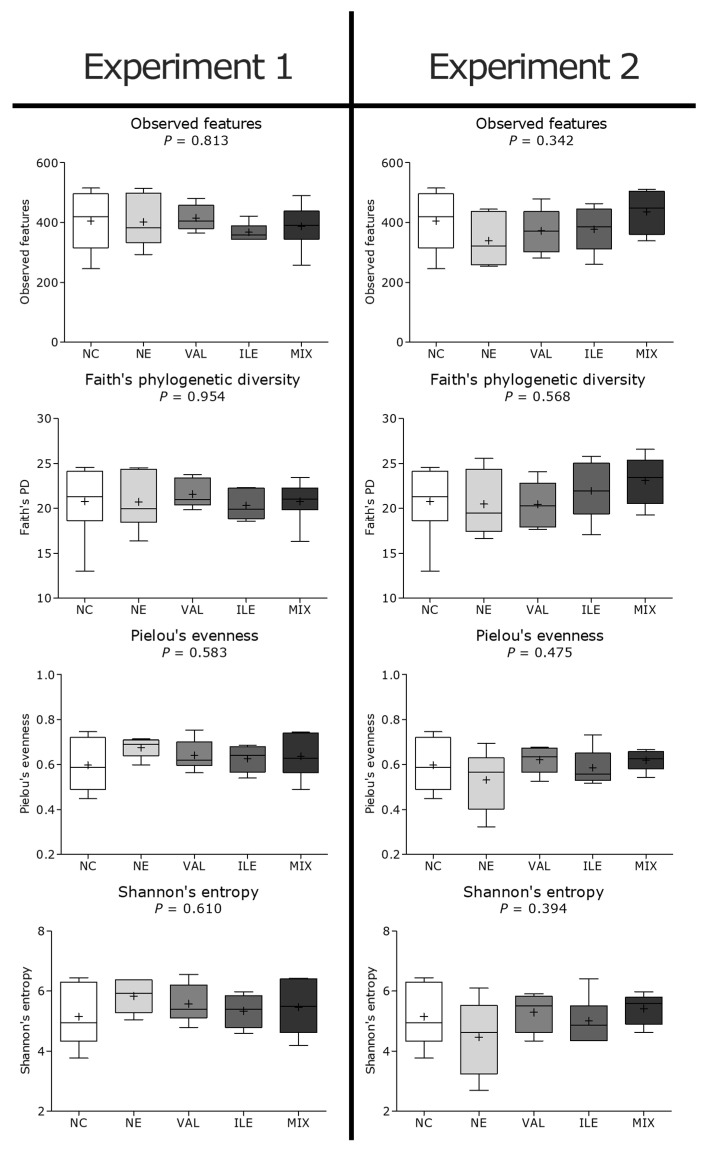
Effects of valine and isoleucine supplementation in two necrotic enteritis challenge experiments on alpha diversity of cecal microbial communities in broilers on d 21 (7 dpi). No significant differences were observed in all alpha diversity indices between the groups (*p* > 0.05). Abbreviations: NC, non-challenged control (leucine/lysine = 1.12; valine/lysine = 0.76; isoleucine/lysine = 0.65, as a calculated value); NE, NE challenge group; VAL, 130% additional valine diet (valine/lysine = 0.98, as a calculated value) in NE-challenged conditions; ILE, 130% additional isoleucine diet (isoleucine/lysine = 0.84, as a calculated value) in NE-challenged conditions; MIX, 130% additional valine and isoleucine diet (valine/lysine = 0.98 and isoleucine/lysine = 0.84, as a calculated value) in NE-challenged conditions. NE challenge in Experiment 1: challenge with 12,500 *E. maxima* oocysts on d 14 followed by *C. perfringens* 1 × 10^9^ challenge on d 18. NE challenge in Experiment 2: challenge with 12,500 *E. maxima* oocysts on d 14 followed by *C. perfringens* 1 × 10^9^ challenge three times on d 18, 19, and 20.

For the statistical analysis, RStudio software (R Version 4.2.2, RStudio PBC, Boston, MA, USA) was used, and the statistical procedure followed a previous study [50]. First, the Shapiro–Wilk test was conducted to test for thew normal distribution of data. If the *p*-value was more than 0.05, Bartlett’s test was then conducted to test homoscedasticity. If the Bartlett’s test result satisfied homoscedasticity (*p* > 0.05), one-way ANOVA was then conducted for data analysis. If the *p*-value of the one-way ANOVA was less than 0.05, Tukey’s honestly significant difference (HSD) post hoc test was performed. For the nonparametric data, such as NE lesion scores, the Kruskal–Wallis test was used, and the Steel–Dwass post hoc test was performed to test the difference between the groups if the *p*-value was less than 0.05. The phylum and family levels were selected to express the relative frequency percentages of cecal bacterial communities, and up to ten items were used for comparison in the order of the highest frequency at each level. Four alpha diversity indices were analyzed, including observed features, Faith’s phylogenetic diversity, Pielou’s evenness, and Shannon’s entropy. Four beta diversity indices, including unweighted UniFrac distance, weighted UniFrac distance, Bray–Curtis dissimilarity, and Jaccard similarity, were analyzed using Principal Coordinate Analysis (PCoA) plots. The permutational multivariate ANOVA (PERMANOVA) test with 999 permutations was used to indicate the difference in beta diversity analysis. Alpha diversity indices were analyzed for all groups within each NE challenge experiment (Figure 3), and beta diversity indices were analyzed in a format comparing differences between the two groups (e.g., NC vs. VAL).

## 3. Results

### 3.1. Growth Performance

No animal mortality was observed during the study. The results of the valine and isoleucine supplementation in two NE challenge experiments on growth performance in broilers from d 7 to 21 are presented in Table 3. In the pre-challenge period (d 7 to 14), no significant differences were observed in growth performance among all groups (*p* > 0.05). In Exp-1, the NC group had significantly higher final BW (d 21) compared to the VAL, ILE, and MIX groups (*p* < 0.001). The ILE group had significantly decreased BW compared to the NE group (*p* < 0.001); it showed the lowest BW on d 21. In the challenge period from d 15 to 21, all NE-challenged groups had significantly decreased BWG compared to the NC group, and the ILE group had significantly reduced BWG compared to the NE group (*p* < 0.001). In the overall period (d 7 to 21), the ILE group also had significantly decreased BWG compared to the NC and NE groups (*p* < 0.001). The ILE and MIX groups had significantly decreased FI compared to the NC group from d 15 to 21 (*p* < 0.01). The ILE group also had significantly lower FI compared to the NC group from d 7 to 21 (*p* < 0.05). In FCR, the ILE group had significantly increased FCR compared to the NC group from d 15 to 21 (*p* < 0.05). In the overall period, the VAL, ILE, and MIX groups had significantly higher FCR compared to the NC group (*p* < 0.01). In Exp-2, again no significant differences were observed in all growth performance among all groups from d 7 to 14 (*p* > 0.05). On d 21 (7 dpi), all NE-challenged groups (NE, VAL, ILE, and MIX) had significantly decreased BW compared to the NC group (*p* < 0.001). In addition, all NE-challenged groups had significantly reduced BWG, FI, and feed efficiency compared to the NC group in both the NE challenge period (d 15 to 21) and overall period (d 7 to 21) (*p* < 0.001). No significant differences were observed in all growth performance measurements among the NE-challenged groups in Exp-2 (*p* > 0.05).

### 3.2. Daily Feed Intake

Daily feed intake was measured from d 8 to 21, and there was no significant difference among all groups prior to the first *C. perfringens* challenge on 4 dpi (d 18; *p* > 0.05). In Exp-1, significant differences were observed on 6 and 7 dpi (d 20 and 21), and all NE-challenged groups had significantly decreased DFI compared to the NC group (*p* < 0.001; Figure 2A). In NE-challenged groups, the ILE group in Exp-1 had significantly lower DFI compared to the NE and VAL groups (*p* < 0.001). In Exp-2, all NE-challenged groups had significantly decreased DFI compared to the NC group on 5, 6, and 7 dpi (*p* < 0.05; Figure 2B). No significant difference was observed in overall DFI in all NE-challenged groups in Exp-2 (*p* > 0.05).

### 3.3. Intestinal Permeability and Jejunal NE Lesion Scores

The results of the valine and isoleucine supplementation in the two NE challenge experiments on intestinal permeability and jejunal NE lesion scores on 7 dpi (d 21) are presented in Table 4. The MIX group had significantly increased intestinal permeability compared to the NC group in Exp-1 (*p* < 0.01). All NE-challenged groups (NE, VAL, ILE, and MIX) also had significantly increased jejunal NE lesion scores compared to the NC group in Exp-1 (*p* < 0.01). In Exp-1, no significant differences were observed among the NE-challenged groups in both intestinal permeability and NE lesion scores (*p* > 0.05). In Exp-2, all NE-challenged groups had significantly increased intestinal permeability and jejunal NE lesion scores compared to the NC group (*p* < 0.01). Similarly to the results of Exp-1, there were no significant differences among all NE-challenged groups in both intestinal permeability and NE lesion scores in Exp-2 (*p* > 0.05).

### 3.4. Jejunal C. perfringens Colony Counts and Fecal E. maxima Oocyst Counts

All NE-challenged groups (NE, VAL, ILE, and MIX) in both NE challenge Exp-1 and Exp-2 had significantly increased jejunal *C. perfringens* colony counts and fecal *E. maxima* oocyst counts compared to the NC group (Table 4; *p* < 0.001). No significant differences were observed among the NE-challenged groups in both jejunal *C. perfringens* colony counts and fecal *E. maxima* oocyst counts (*p* > 0.05).

### 3.5. Jejunal Villus Height: Crypt Depth Ratio and Goblet Cell Count

The results of the valine and isoleucine supplementation in two NE challenge experiments on jejunal VH:CD and intestinal goblet cell counts in broilers on 7 dpi (d 21) are presented in Table 5. In Exp-1, the NE and MIX groups had significantly decreased jejunal villus height compared to the NC group (*p* < 0.05). However, all NE-challenged groups had significantly decreased jejunal VH:CD and goblet cell counts compared to the NC group in Exp-1 (*p* < 0.001). In Exp-2, all NE-challenged groups (NE, VAL, ILE, and MIX) had significantly lower jejunal villus height, VH:CD, and goblet cell counts compared to the NC group (*p* < 0.001). No significant differences were observed in jejunal villus height, VH:CD, and goblet cell counts among the NE-challenged groups in Exp-1 and Exp-2 (*p* > 0.05).

### 3.6. Breast Muscle Weight and Body Fat, Lean, and Mineral Compositions

The results of the valine and isoleucine supplementation in two NE challenge experiments on breast muscle yield and body composition using DEXA on d 21 (7 dpi) are presented in Table 6. No significant difference was observed in breast muscle weight and relative breast muscle weight among the groups in Exp-1 and Exp-2 (*p* > 0.05). In Exp-1, the ILE group had significantly lower total tissue weight and lean weight compared to the NC group (*p* < 0.05). The ILE group also had significantly reduced BMC compared to the NC group (*p* < 0.05). However, no significant differences were observed among all NE-challenged groups in all measurements using DEXA in Exp-1 (*p* > 0.05). In Exp-2, all NE-challenged groups had significantly decreased total tissue weight compared to the NC group (*p* < 0.05). In addition, the NE group had significantly lower lean weight compared to the NC group in Exp-2 (*p* < 0.05). The NE, ILE, and MIX groups had significantly reduced BMC compared to the NC group; however, the VAL group had similar BMC compared to the NC group in Exp-2 (*p* < 0.01). Similarly to the results of Exp-1, no significant differences were observed among all NE-challenged groups in all measurements using DEXA in Exp-2 (*p* > 0.05).

### 3.7. Jejunal Gene Expression Levels

The results of the valine and isoleucine supplementation in two NE challenge experiments on jejunal gene expression levels in broilers on 7 dpi (d 21) are presented in Table 7. In Exp-1, all NE-challenged groups had significantly increased *L-type AA transporter 1* (*LAT1*) gene expression levels compared to the NC group (*p* < 0.001). The MIX group had significantly higher *sodium-coupled neutral AA transporter 2* (*SNAT2*) gene expression levels compared to the NC group (*p* < 0.01). All NE-challenged groups had significantly decreased expression levels of the *sodium-independent AA transporter 1* (*BAT1*) gene compared to the NC group (*p* < 0.001). The NE group also had significantly increased expression levels of *interleukin 1 beta* (*IL1β*), *IL 1 receptor antagonist* (*IL1RN*), *IL10*, *interferon gamma* (*IFN-γ*), *C-C motif chemokine ligand 4* (*CCL4*), *C-X-C motif chemokine ligand 8* (*CXCL8*), *TLR2, TLR4*, and *NFκB1* genes compared to the NC group (*p* < 0.05). The VAL, ILE, and MIX groups had significantly increased expression levels of *IL1RN*, *IL10*, *IFN-γ*, *CCL4*, and *NFκB1* genes compared to the NC group (*p* < 0.01). The VAL and MIX groups had significantly increased *IL6* gene expression levels compared to the NC group (*p* < 0.01). The ILE and MIX groups had significantly increased *TLR2* gene expression levels compared to the NC group (*p* < 0.01). The ILE group had significantly increased *TLR4* gene expression levels compared to the NC group (*p* < 0.05). However, no significant differences were observed in all jejunal gene expression levels among all NE-challenged groups (*p* > 0.05).

In Exp-2, all NE-challenged groups had significantly higher expression levels of *LAT1* and *SNAT2* genes compared to the NC group (*p* < 0.01). Similarly to the result of Exp-1, all NE-challenged groups also had significantly decreased *BAT1* gene expression levels compared to the NC group (*p* < 0.001). All NE-challenged groups (NE, VAL, ILE, and MIX) had significantly increased expression levels of *IL1RN*, *IL10*, *IFN-γ*, and *CCL4* genes compared to the NC group (*p* < 0.01). The VAL, ILE, and MIX groups had significantly increased *IL1β* gene expression levels compared to the NC group (*p* < 0.05). The NE, VAL, and MIX groups had significantly increased *IL6* gene expression levels compared to the NC group (*p* < 0.01). In addition, the groups of NE, ILE, and MIX had significantly increased *CXCL8* gene expression levels compared to the NC group (*p* < 0.05). Both the ILE and MIX groups had significantly increased expression levels of *TLR2* and *NFκB1* genes compared to the NC group (*p* < 0.05). Similarly to the Exp-1 results, no significant differences were observed in all jejunal gene expression levels among all NE-challenged groups (*p* > 0.05).

### 3.8. Breast Muscle Gene Expression Levels

The results of the valine and isoleucine supplementation in two NE challenge experiments on breast muscle gene expression levels in broilers on 7 dpi (d 21) are presented in Table 8. In Exp-1, the NE and ILE groups had significantly decreased expression levels of *ribosomal protein S6 kinase B1* (*S6K1*) and *protein phosphatase Mg^2+^/Mn^2+^ dependent 1K* (*PPM1K*) genes compared to the NC group (*p* < 0.01). In addition, both NE and ILE groups had significantly increased *BCAA transaminase 1* (*BCAT1*) gene expression levels compared to the MIX group (*p* < 0.01). The ILE group had significantly decreased *LAT1* gene expression levels compared to the NC and VAL groups (*p* < 0.01). Furthermore, the ILE group had significantly decreased *SNAT2* gene expression levels compared to the NC group (*p* < 0.01).

In Exp-2, the NC and VAL groups had significantly increased *BCAT1* gene expression levels compared to the MIX group (*p* < 0.01). Both the NE and MIX groups had significantly decreased *PPM1K* gene expression levels compared to the NC group (*p* < 0.01). In addition, the VAL, ILE, and MIX groups had significantly decreased *LAT1* gene expression levels compared to the NC group (*p* < 0.01). The NE group had the significantly highest *LAT4* gene expression levels compared to all other groups (*p* < 0.001). The ILE and MIX groups had significantly lower *SNAT2* gene expression levels compared to the NC group (*p* < 0.05).

### 3.9. Taxonomic Composition of Cecal Microbial Communities

The results of the valine and isoleucine supplementation in two NE challenge experiments on phylum-level relative frequency of cecal bacterial communities in broilers on 7 dpi are presented in Table 9. In both NE Exp-1 and Exp-2, no significant differences were observed in all phylum-level relative frequencies among the groups (*p* > 0.05).

The results of the valine and isoleucine supplementation in two NE challenge experiments on family-level relative frequency of cecal bacterial communities in broilers on 7 dpi are presented in Table 10. Similarly to the results of the phylum-level relative frequencies, there were no significant differences among the groups all family-level relative frequencies among the groups (*p* > 0.05).

### 3.10. Alpha and Beta Diversities of Cecal Microbial Communities

The results of the valine and isoleucine supplementation in two NE challenge experiments on alpha diversity of cecal bacterial communities in broilers on 7 dpi are presented in Figure 3. No significant differences were observed in all alpha diversity indices among all groups in both NE challenge experiments (*p* > 0.05).

**Figure 4:**
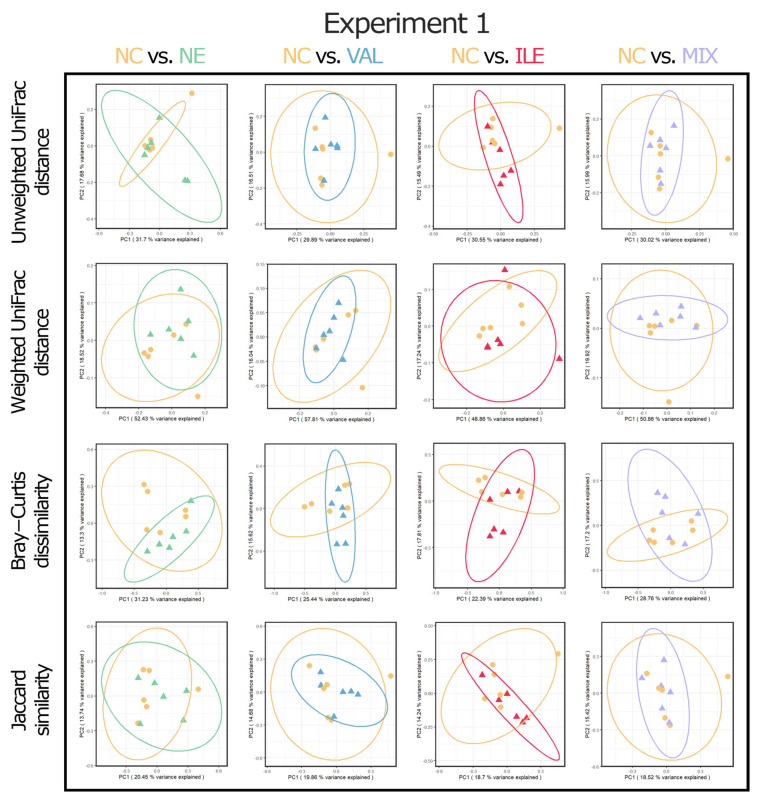
Effects of valine and isoleucine supplementation in necrotic enteritis challenge Experiment 1 on beta diversity of cecal microbial communities in broilers on d 21 (7 dpi). No significant differences were observed in all alpha diversity indices between the groups. Abbreviations: NC, non-challenged control (leucine/lysine = 1.12; valine/lysine = 0.76; isoleucine/lysine = 0.65, as a calculated value); NE, NE challenge group; challenge with 12,500 *E. maxima* oocysts on d 14 followed by *C. perfringens* 1 × 10^9^ challenge on d 18; VAL, 130% additional valine diet (valine/lysine = 0.98, as a calculated value) in NE-challenged conditions; ILE, 130% additional isoleucine diet (isoleucine/lysine = 0.84, as a calculated value) in NE-challenged conditions; MIX, 130% additional valine and isoleucine diet (valine/lysine = 0.98 and isoleucine/lysine = 0.84, as a calculated value) in NE-challenged conditions.

**Figure 5:**
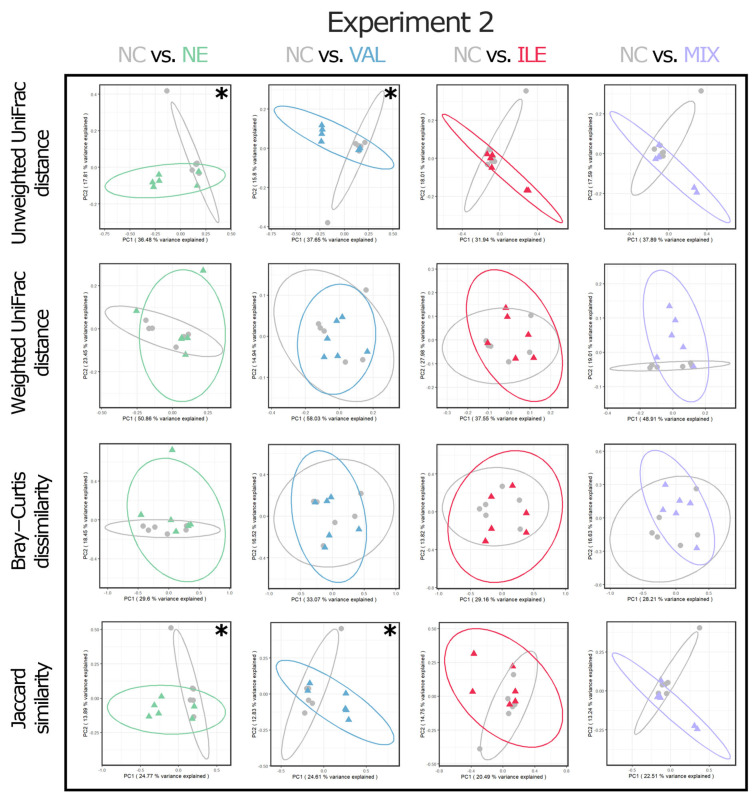
Effects of valine and isoleucine deficiency in necrotic enteritis challenge Experiment 2 on beta diversity of cecal microbial communities in broilers on d 21 (7 dpi). Abbreviations: NC, non-challenged control (leucine/lysine = 1.12; valine/lysine = 0.76; isoleucine/lysine = 0.65, as a calculated value); NE, NE challenge group; challenge with 12,500 *E. maxima* oocysts on d 14 followed by *C. perfringens* 1 × 10^9^ challenge three times on d 18, 19, and 20; VAL, 130% additional valine diet (valine/lysine = 0.98, as a calculated value) in NE-challenged conditions; ILE, 130% additional isoleucine diet (isoleucine/lysine = 0.84, as a calculated value) in NE-challenged conditions; MIX, 130% additional valine and isoleucine diet (valine/lysine = 0.98 and isoleucine/lysine = 0.84, as a calculated value) in NE-challenged conditions. *, *p* < 0.05.

The results of the valine and isoleucine supplementation in two NE challenge experiments on beta diversity PCoA plots of cecal bacterial communities are presented in Figure 4 and Figure 5. In Exp-1, no significant differences were observed in all beta diversity indices among all groups (Figure 4; *p* > 0.05).

In Exp-2, however, both the NE and VAL groups showed significant differences in unweighted UniFrac distance and Jaccard similarity compared to the NC group (Figure 5; *p* < 0.05).

## 4. Discussion

The aim of the current study was to test the extent to which additional BCAAs can the alleviate negative effects of NE infection, and two different severities of NE challenges were established to explore the effects of additional BCAAs under more diverse NE challenge conditions. The additional BCAA levels (130%) in the current study were based on the previous dose–response study, as the addition of 125% valine to the high leucine diet had the highest BWG and feed efficiency [55]. Based on the previous results, the 130% level was established in the current study based on the hypothesis that higher AA levels may be required due to increased energy consumption in NE-challenged conditions [56].

Significant BWG reductions in the NE-challenged groups (NE, VAL, ILE, and MIX) due to the NE challenge were observed during the challenge period (d 15 to 21) in both experiments. This indicates that NE challenge in the current study, which was established based on our previous NE coinfection model, effectively deteriorated growth performance [18,37,43]. Additional BCAA groups (VAL, ILE, and MIX) did not mitigate growth reduction due to the NE infection in any NE challenge experiments. In particular, additional isoleucine in Exp-1 resulted in further deterioration of growth performance. On the other hand, in Exp-2, no further negative effect due to additional isoleucine was observed. The reduced growth performance from the additional isoleucine diet likely affected the availability of valine from a BCAA antagonistic perspective in broilers [57]. Previous studies have reported that excessive dietary isoleucine levels [58,59] or valine deficiency can have negative effects on chicken growth [43,47]. However, it is noteworthy that the response to additional BCAAs in the diet varies depending on the intensity of NE challenges. This is because the NE infection significantly reduces feed consumption, so the dietary effects can change depending on the intensity of NE. Goo et al. (2025) reported that when NE infection and valine deficiency occur together, the effect of NE infection can be more significant than the dietary effect in the 5 to 7 dpi when the infection effect is most pronounced [43]. During this period, NE infection generated a negative effect regardless of the effectiveness of the diet. Therefore, the negative effects of additional isoleucine observed in the relatively mild NE challenge experiment (Exp-1) may have been due to the relatively sufficient time for the dietary factor to be reapplied compared to the severe NE challenge experiment (Exp-2) in the current study. These results can be seen in the DFI graph (Figure 2), where all NE-challenged groups in Exp-2 continued to decrease feed consumption until the end of the study (7 dpi), whereas the NE and VAL groups in Exp-1 did not decrease further at 7 dpi. However, it is clear that the effect of the additional BCAAs occurred only with the additional isoleucine, whereas the additional valine did not show a significant change. It has been previously reported that adding additional valine to the normal diet does not exhibit a significant beneficial effect in broilers [33,60,61], and the current study confirmed that it exhibits a similar response under NE infection conditions. However, because the current study was conducted until d 21 (7 dpi), further studies are needed to determine the significant effect of additional BCAAs in the subsequent recovery phase.

There were no significant differences in intestinal permeability, jejunal NE lesion scores and *C. perfringens* colony counts, and fecal *E. maxima* oocyst counts among the NE-challenged groups (NE, VAL, ILE, and MIX) in both NE challenge experiments (Table 4). Furthermore, similar results among the NE-challenged groups were observed in the jejunal histology parameters, including villus height, VH:CD, and goblet cell counts (Table 5). It is well known that NE coinfection using *E. maxima* and *C. perfringens* exacerbates overall intestinal health in broilers, and similar results have been observed in the current study [18,37,43]. Both jejunal *C. perfringens* colony counts and fecal *E. maxima* oocyst counts were higher than those of the non-challenged group, and the increased jejunal NE lesion score also indicated that the NE challenge in the current study was successfully developed in the jejunum. Significant reductions in jejunal villus height, VH:CD, and goblet cells also indicated that the NE coinfection model using *E. maxima* and *C. perfringens* resulted in a decreased capability of nutrient uptake and mucin secretion [62]. As such, the deterioration of intestinal health, intestinal morphology, and decreased mucin secretion may significantly reduce nutrient and AA uptake capacity and cause the host to be more susceptible to external pathogens, leading to a significant decrease in chicken growth [63,64]. However, differences were observed between the groups in intestinal permeability measurements depending on the NE challenge experiments. This is similar to previous studies; intestinal permeability analysis using serum FITC-d may not show a statistical difference between the non-challenged control groups and the mild or weak NE-challenged groups, despite up to a 70-fold difference between the groups due to high measurement deviation [18,37,42]. Overall intestinal health was severely deteriorated in both NE challenges, and none of the additional BCAA groups (VAL, ILE, and MIX) alleviated the damaged intestinal integrity from the NE challenge. Notably, the ILE group in Exp-1 further decreased growth performance, but no such differences were found in any intestinal health measurements. This result agrees with a previous study by Goo et al. (2025b), indicating that the effects of BCAA-level changes in the diet may be applied independently of intestinal health [43].

The negative effects of additional isoleucine were similarly observed in body composition results in Exp-1 (Table 6). Additional isoleucine in Exp-1 showed the lowest total tissue and lean weights. This is similar to the results of d 21 BW, which may be the result of increased isoleucine causing BCAA antagonism, affecting the availability of valine and thus failing to achieve adequate growth. In Exp-2, all NE-challenged groups showed reduced total tissue weight compared to the non-challenged group. NE challenge was observed to severely reduce total tissue weight, lean weight, and BMC, even in the relatively weak NE challenge (Exp-1), which may be the result of severe intestinal damage caused by *C. perfringens* interfering with nutrient absorption and digestion [2]. The notable point in the DEXA analysis in the current study is the difference in BMC levels. In particular, in Exp-2, only the additional valine group had increased BMC levels despite the decreased total tissue weight. The decreased BMC levels may be attributed to the reduced nutrient absorption capacity and digestibility caused by the NE challenge [37,65], resulting in a similar response to that of AA deficiency [43]. Because dietary valine levels play an important role in bone calcification and in the regulation of calcium levels in chickens [33,66,67], it is likely that additional valine in the current study restored BMC levels reduced by severe NE challenge. Thus, although additional BCAAs did not significantly restore BW and muscle weight loss caused by NE infection, additional valine may potentially improve some of the bone health deterioration in broilers under different NE severity conditions.

Gene expression analysis was performed to investigate how additional BCAAs under the NE-challenged conditions are potentially related to the activities of various AA transporters and immune responses in the jejunum on d 21. Both NE challenges upregulated jejunal *LAT1* gene expression levels but downregulated *BAT1* expression levels (Table 7). In addition, the NE challenge upregulated most of the expression levels of inflammatory cytokine and chemokine genes regardless of the intensity of NE challenges. Pathogen recognition receptors such as TLRs and *NFκB1* genes were also included in the assay category, and their expression levels were upregulated in the NE challenge. This is because innate immune responses and cytokine secretion are initiated by TLR2 and TLR4 in response to pathogen-associated molecular patterns of external bacteria, such as *C. perfringens* [68,69]. Particularly, the increased expression levels of *LAT1*, *IL10*, *IFN-γ*, and *CCL4* were noticeable in both NE challenges, with differences ranging from a minimum of 4.6 to a maximum of 21.6-fold compared to the non-challenged control group. This result may be attributed to the NetB produced by type G *C. perfringens*, highly proliferated under the *E. maxima* coinfection condition in the current study, which triggers an innate and adaptive immune response with the direct disruption of jejunal epithelial cells. The induction of the immune response by NetB may have activated more cytokines and chemokines, thereby attracting various immune cells, such as T cells, NK cells, and neutrophils, to the NE infection site [70,71,72]. Noteworthy, the opposite pattern was observed in AA transporter gene expression levels, where *LAT1* was upregulated up to 11-fold, whereas *BAT1* was downregulated up to 5-fold compared to the non-challenged control. Reduced gene expression of *BAT1* was likely caused by physically damaged jejunal epithelial cells due to *Eimeria* reproduction [73,74,75], which in turn may have affected the brush border membrane transport ability of neutral and cationic AAs [76]. On the other hand, the increased expression level of the *LAT1* gene in the NE-challenged conditions was also observed in *Eimeria* and *C. perfringens* challenge studies [43,77,78], which may have been affected by activated T cells and NK cells migrating to the jejunal area due to acute immune responses [79,80,81]. In addition, since the *SNAT2* also plays a crucial role in glutamine uptake by T cells [82,83], it may consequently suggest that the activity of T cells by NE infection may be significantly increased in the current study. Although NE infection caused widespread and excessive immune responses in the jejunum, none of the added BCAA groups effectively alleviated it.

Breast muscle gene expression levels showed relatively fewer differences between the groups compared to gene expression in the jejunum. Nevertheless, unlike jejunal gene expression levels, differences were observed between the BCAA groups in the breast muscle. However, the differences between the groups varied depending on the intensity of the NE challenge; in Exp-1, the MIX group (additional valine and isoleucine) increased *BCAT1* gene expression levels compared to the NE and ILE groups, whereas in Exp-2, the MIX group showed the lowest gene expression level (Table 8). In addition, differences in *LAT1* gene expression levels were found between the additional valine and isoleucine groups only in Exp-1. In Exp-2, only the NE challenge group showed the highest *LAT4* gene expression levels, but there was no significant difference among the groups in Exp-1. The increased levels of *BCAT1* and *LAT1* expression in the additional valine group in Exp-1 could be attributed to the fact that valine is the most important and efficient AA in producing energy through the TCA cycle [27,81]. Therefore, if the excessive immune response due to NE infection further increased the energy requirement in the chickens [56,84], BCAA catabolism enzyme activity may have increased as a way to compensate for this. Decreased *PPM1K* gene expression in the NE group was observed compared to the NC group in both NE challenges, but no difference was found among the NE-challenged groups. Protein phosphatase Mg^2+^/Mn^2+^ dependent 1K is an endogenous phosphatase of the branched-chain α-keto acid dehydrogenase (BCKDH) complex, a key enzyme in BCAA catabolic steps in animals, and is known to regulate BCAA homeostasis and degradation [85,86]. The decreased expression of *PPM1K* may indicate that BCAA utilization and degradation capacity can be reduced under NE infection in chickens; *PPM1K* can act more sensitively than the BCKDH kinase (BCKDK) and BCKDH enzymes involved in BCAA catabolism do. On the other hand, this difference in BCAA enzyme activity did not lead to differences in d 21 growth performance among the additional BCAA groups (VAL, ILE, and MIX), indicating that the dietary leucine level in the current experiment, which is a recommended proper leucine level (leucine/lysine = 1.12), might be more responsible. It consequently means that a well-balanced BCAA diet was provided to the chickens in the current study. Therefore, it is likely that additional valine or/and isoleucine in a balanced BCAA ratio diet caused antagonistic effects. This indicates that growth performance in broilers cannot be easily improved without considering BCAA ratios together, suggesting that even under a NE challenge, an appropriate BCAA ratio may need to be maintained.

Similarly to the previously reported studies [43,47,50,57], dietary BCAA levels and cecal microbial communities do not have meaningful relationships in all cecal microbiome analyses (Table 9 and Table 10). Although the additional valine in Exp-2 showed differences in beta diversity indices, including unweighted UniFrac distance and Jaccard similarity, compared to the non-challenged group (Figure 5), there was no significant difference among the BCAA groups. Additional BCAA in the diet under NE-challenged conditions has no significant effect on the cecal microbial community; consequently, it is thought that there is less association with overall intestinal health in broilers.

## 5. Conclusions

In conclusion, broiler growth was significantly affected by the NE challenge. NE infection can deteriorate overall intestinal health and trigger complex immune responses, severely impeding the growth efficiency of chickens. However, any additional BCAAs did not significantly improve growth performance in broilers fed a BCAA-balanced diet, regardless of the intensity of the NE challenge. In the relatively weak NE challenge experiment (Exp-1), the negative effects of additional isoleucine were compounded by the NE challenge effects. In addition, unlike the additional valine groups (VAL and MIX), 130% additional isoleucine hardly changed the expression levels of protein synthesis, BCAA catabolism, and AA transporter-related genes in breast muscle. However, these gene expression levels changed with the intensity of the NE challenges. In the relatively severe NE challenge experiment (Exp-2), additional BCAAs had no significant effects on growth performance, and no further reductions occurred. However, additional valine alleviated the bone mineral content loss from NE infection. No significant differences in intestinal permeability, jejunal lesion score and *C. perfringens* colony counts, fecal *E. maxima* oocyst counts, jejunal VH:CD and goblet cell counts, jejunal gene expression levels, breast muscle yield, body mineral compositions, and cecal microbiome were observed among the NE-challenged groups (NE, VAL, ILE, and MIX) regardless of the intensity of the NE challenge. Consequently, additional BCAAs are considered to have no effect in terms of improving intestinal health under NE conditions, but the changes in growth performance and bone mineral contents due to additional BCAAs depending on the intensity of the NE challenge require further research.

## Figures and Tables

**Table 1:** Diet composition of the current study from d 7 to 21 (as-fed basis, %).

	Treatments ^1^			
	NC or NE	NE-Challenged		
		VAL	ILE	MIX
Ingredient, %				
Corn, grain	65.75	65.75	65.75	65.75
Soybean meal, 46.9% CP	25.75	25.75	25.75	25.75
Soybean oil	1.25	1.25	1.25	1.25
Dicalcium phosphate	1.60	1.60	1.60	1.60
Limestone	1.20	1.20	1.20	1.20
Sodium bicarbonate	0.35	0.35	0.35	0.35
Salt	0.22	0.22	0.22	0.22
DL-Methionine	0.45	0.45	0.45	0.45
L-Lysine HCl	0.42	0.42	0.42	0.42
L-Threonine	0.28	0.28	0.28	0.28
L-Arginine	0.33	0.33	0.33	0.33
L-Valine	0.21	0.21	0.21	0.48
L-Isoleucine	0.11	0.11	0.11	0.36
Glycine	1.55	1.37	1.40	1.23
Sand	0.35	0.26	0.25	0.15
Vitamin premix ^2^	0.10	0.10	0.10	0.10
Mineral premix ^3^	0.08	0.08	0.08	0.08
Total	100.0	100.0	100.0	100.0
Calculated value, %				
ME, kcal/kg	3000	3000	3000	3000
Crude protein	20.0	20.0	20.0	20.0
Total calcium	0.88	0.88	0.88	0.88
Available phosphorus	0.44	0.44	0.44	0.44
Arginine	1.29	1.29	1.29	1.29
Lysine	1.17	1.17	1.17	1.17
Methionine	0.68	0.68	0.68	0.68
TSAA	0.89	0.89	0.89	0.89
Threonine	0.80	0.80	0.80	0.80
Tryptophan	0.18	0.18	0.18	0.18
Leucine	1.31	1.31	1.31	1.31
Valine	0.89	1.15	0.89	1.15
Isoleucine	0.76	0.76	0.98	0.98
BCAA/lysine ratio				
Leucine/lysine	1.12	1.12	1.12	1.12
Valine/lysine	0.76	0.98	0.76	0.98
Isoleucine/lysine	0.65	0.65	0.84	0.84

^1^ NC, non-challenged control (leucine/lysine = 1.12; valine/lysine = 0.76; isoleucine/lysine = 0.65); NE, NE challenge with 12,500 *E. maxima* oocysts on d 14 followed by *C. perfringens* 1 × 10^9^ challenge on d 18 or challenge with 12,500 *E. maxima* oocysts on d 14 followed by *C. perfringens* 1 × 10^9^ challenge three times on d 19, 20, and 21; VAL, 130% additional valine diet (valine/lysine = 0.98) in NE-challenged conditions; ILE, 130% additional isoleucine diet (isoleucine/lysine = 0.84) in NE-challenged conditions; MIX, 130% additional valine and isoleucine diet (valine/lysine = 0.98 and isoleucine/lysine = 0.84) in NE-challenged conditions. ^2^ Vitamin premix provided the following per kg of diet: vitamin A, 3527 IU; vitamin D3, 1400 IU; vitamin E, 19.4 IU; niacin, 20.28 mg; D-pantothenic acid, 5.47 mg; riboflavin, 3.53 mg; vitamin B6, 1.46 mg; menadione, 1.10 mg; thiamin, 0.97 mg; folic acid, 0.57 mg; biotin, 0.08 mg; vitamin B12, 0.01 mg. ^3^ Mineral premix provided the following per kg of diet: Mn, 100.5 mg; Zn, 80.3 mg; Ca, 24 mg; Mg, 20.1 mg; Fe, 19.7 mg; Cu, 3 mg; I, 0.75 mg; Se, 0.30 mg.

**Table 2:** Analyzed diet composition of the current study (as-fed basis, %).

	Treatments ^1^			
	NC or NE	NE-Challenged		
		VAL	ILE	MIX
Analyzed value, %				
Alanine	0.79	0.87	0.94	0.85
Arginine	1.32	1.42	1.43	1.37
Aspartic Acid	1.56	1.78	1.94	1.72
Cysteine	0.23	0.28	0.30	0.26
Glutamic Acid	2.77	3.16	3.43	3.04
Glycine	1.85	1.63	1.61	1.40
Histidine	0.39	0.44	0.48	0.42
Isoleucine	0.83	0.92	1.19	1.16
Leucine	1.36	1.50	1.62	1.50
Lysine	1.18	1.28	1.30	1.23
Methionine	0.57	0.67	0.64	0.59
Phenylalanine	0.78	0.87	0.94	0.86
Proline	0.88	0.97	1.05	0.96
Serine	0.63	0.73	0.78	0.70
Threonine	0.86	0.87	0.92	0.80
Tryptophan	0.20	0.19	0.21	0.20
Tyrosine	0.53	0.59	0.63	0.62
Valine	0.97	1.37	1.09	1.28
Crude protein, %	19.6	20.1	20.1	20.4
BCAA/lysine ratio				
Leucine/lysine	1.15	1.17	1.25	1.22
Valine/lysine	0.82	1.07	0.84	1.04
Isoleucine/lysine	0.70	0.72	0.92	0.94

^1^ NC, non-challenged control (leucine/lysine = 1.12; valine/lysine = 0.76; isoleucine/lysine = 0.65, as a calculated value); NE, NE challenge with 12,500 *E. maxima* oocysts on d 14 followed by *C. perfringens* 1 × 10^9^ challenge on d 18 or challenge with 12,500 *E. maxima* oocysts on d 14 followed by *C. perfringens* 1 × 10^9^ challenge three times on d 19, 20, and 21; VAL, 130% additional valine diet (valine/lysine = 0.98, as a calculated value) in NE-challenged conditions; ILE, 130% additional isoleucine diet (isoleucine/lysine = 0.84, as a calculated value) in NE-challenged conditions; MIX, 130% additional valine and isoleucine diet (valine/lysine = 0.98 and isoleucine/lysine = 0.84, as a calculated value) in NE-challenged conditions.

**Table 3:** Effects of valine and isoleucine supplementation in two necrotic enteritis challenge experiments on growth performance in broilers from d 7 to 21.

	NC ^1^	NE Challenge				SEM (*n* = 6)	*p*-Value
		NE	VAL	ILE	MIX		
Experiment 1 ^2^							
Body weight, g							
d 7	160	159	159	159	159	0.49	0.988
d 14 (0 dpi ^3^)	450	461	452	447	454	7.8	0.770
d 21 (7 dpi)	864 ^a^	813 ^ab^	796 ^bc^	749 ^c^	788 ^bc^	14.1	<0.001
Body weight gain, g							
d 7–14	291	302	293	287	295	7.7	0.750
d 15–21	414 ^a^	353 ^b^	344 ^bc^	302 ^c^	333 ^bc^	11.3	<0.001
d 7–21	704 ^a^	654 ^ab^	637 ^bc^	589 ^c^	628 ^bc^	14.0	<0.001
Feed intake, g							
d 7–14	343	354	354	351	350	5.5	0.661
d 15–21	572 ^a^	533 ^ab^	529 ^ab^	488 ^b^	517 ^b^	12.5	<0.01
d 7–21	915 ^a^	887 ^ab^	882 ^ab^	839 ^b^	867 ^ab^	16.0	<0.05
Feed conversion ratio							
d 7–14	1.18	1.17	1.21	1.22	1.19	0.017	0.270
d 15–21	1.39 ^a^	1.51 ^ab^	1.54 ^ab^	1.63 ^b^	1.55 ^ab^	0.044	<0.05
d 7–21	1.30 ^a^	1.36 ^ab^	1.39 ^b^	1.43 ^b^	1.38 ^b^	0.019	<0.01
Experiment 2							
Body weight, g							
d 7	160	159	159	159	159	0.49	0.992
d 14 (0 dpi)	450	450	459	449	457	5.8	0.637
d 21 (7 dpi)	864 ^a^	746 ^b^	767 ^b^	745 ^b^	742 ^b^	13.6	<0.001
Body weight gain, g							
d 7–14	291	290	300	290	298	5.7	0.620
d 15–21	414 ^a^	296 ^b^	308 ^b^	296 ^b^	286 ^b^	12.3	<0.001
d 7–21	704 ^a^	586 ^b^	607 ^b^	586 ^b^	583 ^b^	13.5	<0.001
Feed intake, g							
d 7–14	343	339	352	349	352	3.6	0.062
d 15–21	572 ^a^	482 ^b^	495 ^b^	489 ^b^	489 ^b^	9.5	<0.001
d 7–21	915 ^a^	821 ^b^	847 ^b^	838 ^b^	841 ^b^	11.0	<0.001
Feed conversion ratio							
d 7–14	1.18	1.17	1.18	1.21	1.18	0.016	0.566
d 15–21	1.39 ^a^	1.63 ^b^	1.62 ^b^	1.67 ^b^	1.72 ^b^	0.046	<0.001
d 7–21	1.30 ^a^	1.40 ^b^	1.40 ^b^	1.43 ^b^	1.44 ^b^	0.019	<0.001

^a–c^ Means in the same row with different superscripts are statistically different (*p*-value < 0.05). ^1^ NC, non-challenged control (leucine/lysine = 1.12; valine/lysine = 0.76; isoleucine/lysine = 0.65, as a calculated value). ^2^ NE challenge in Exp-1, challenge with 12,500 *E. maxima* oocysts on d 14 followed by *C. perfringens* 1 × 10^9^ challenge on d 18; NE challenge in Exp-2, challenge with 12,500 *E. maxima* oocysts on d 14 followed by *C. perfringens* 1 × 10^9^ challenge three times on d 19, 20, and 21; VAL, 130% additional valine diet (valine/lysine = 0.98, as a calculated value) in NE-challenged conditions; ILE, 130% additional isoleucine diet (isoleucine/lysine = 0.84, as a calculated value) in NE-challenged conditions; MIX, 130% additional valine and isoleucine diet (valine/lysine = 0.98 and isoleucine/lysine = 0.84, as a calculated value) in NE-challenged conditions. ^3^ dpi, day post inoculation of 12,500 *E. maxima* oocysts on d 14.

**Table 4:** Effects of valine and isoleucine supplementation in two necrotic enteritis challenge experiments on intestinal permeability, jejunal NE lesion score, jejunal *C. perfringens* colony count, and fecal *E. maxima* oocyst count in broilers on d 21 (7 dpi).

	NC ^1^	NE Challenge				SEM (*n* = 6)	*p*-Value
		NE	VAL	ILE	MIX		
Experiment 1 ^2^							
Intestinal permeability, ng/mL	14 ^b^	183 ^ab^	254 ^ab^	263 ^ab^	404 ^a^	67.4	<0.01
Jejunal NE lesion score, 0 to 3 scale	0 ^b^	1.5 ^a^	1.3 ^a^	1.5 ^a^	1.0 ^a^	0.16	<0.01 ^†^
Jejunal *C. perfringens* colony count, log_10_ cfu/g	6.8 ^b^	8.3 ^a^	8.2 ^a^	8.4 ^a^	8.1 ^a^	0.25	<0.001
Fecal *E. maxima* oocyst count, log_10_/g	0 ^b^	5.6 ^a^	5.6 ^a^	5.8 ^a^	5.7 ^a^	0.07	<0.001
Experiment 2							
Intestinal permeability, ng/mL	14 ^b^	273 ^a^	242 ^a^	483 ^a^	287 ^a^	61.5	<0.001
Jejunal NE lesion score, 0 to 3 scale	0 ^b^	1.5 ^a^	1.3 ^a^	1.5 ^a^	1.5 ^a^	0.23	<0.01 ^†^
Jejunal *C. perfringens* colony count, log_10_ cfu/g	6.8 ^b^	8.6 ^a^	8.3 ^a^	8.7 ^a^	8.5 ^a^	0.30	<0.001
Fecal *E. maxima* oocyst count, log_10_/g	0 ^b^	5.7 ^a^	5.7 ^a^	5.7 ^a^	5.9 ^a^	0.08	<0.001

^a,b^ Means in the same row with different superscripts are statistically different (*p*-value < 0.05). ^1^ NC, non-challenged control (leucine/lysine = 1.12; valine/lysine = 0.76; isoleucine/lysine = 0.65, as a calculated value). ^2^ NE challenge in Exp-1, challenge with 12,500 *E. maxima* oocysts on d 14 followed by *C. perfringens* 1 × 10^9^ challenge on d 18; NE challenge in Exp-2, challenge with 12,500 *E. maxima* oocysts on d 14 followed by *C. perfringens* 1 × 10^9^ challenge three times on d 19, 20, and 21; VAL, 130% additional valine diet (valine/lysine = 0.98, as a calculated value) in NE-challenged conditions; ILE, 130% additional isoleucine diet (isoleucine/lysine = 0.84, as a calculated value) in NE-challenged conditions; MIX, 130% additional valine and isoleucine diet (valine/lysine = 0.98 and isoleucine/lysine = 0.84, as a calculated value) in NE-challenged conditions. ^†^ Kruskal–Wallis test and Steel–Dwass post hoc test were performed to test the difference in nonparametric data. Data are expressed as the median.

**Table 5:** Effects of valine and isoleucine supplementation in two necrotic enteritis challenge experiments on jejunal histology and goblet cell count in broilers on d 21 (7 dpi).

	NC ^1^	NE Challenge				SEM (*n* = 6)	*p*-Value
		NE	VAL	ILE	MIX		
Experiment 1 ^2^							
Villus height, μm	1205 ^a^	863 ^b^	923 ^ab^	880 ^ab^	851 ^b^	78.3	<0.05
Crypt depth, μm	168 ^b^	261 ^a^	261 ^a^	266 ^a^	310 ^a^	17.8	<0.001
VH:CD ^3^	7.5 ^a^	3.4 ^b^	3.5 ^b^	3.3 ^b^	2.8 ^b^	0.35	<0.001
Goblet cells, cells/villus	152 ^a^	94 ^b^	96 ^b^	99 ^b^	83 ^b^	10.5	<0.001
Experiment 2							
Villus height, μm	1205 ^a^	733 ^b^	913 ^b^	824 ^b^	856 ^b^	57.9	<0.001
Crypt depth, μm	168 ^b^	272 ^a^	307 ^a^	277 ^a^	293 ^a^	18.5	<0.001
VH:CD	7.5 ^a^	2.8 ^b^	3.0 ^b^	3.0 ^b^	2.9 ^b^	0.33	<0.001
Goblet cells, cells/villus	152 ^a^	78 ^b^	109 ^b^	93 ^b^	104 ^b^	7.8	<0.001

^a,b^ Means in the same row with different superscripts are statistically different (*p*-value < 0.05). ^1^ NC, non-challenged control (leucine/lysine = 1.12; valine/lysine = 0.76; isoleucine/lysine = 0.65, as a calculated value). ^2^ NE challenge in Exp-1, challenge with 12,500 *E. maxima* oocysts on d 14 followed by *C. perfringens* 1 × 10^9^ challenge on d 18; NE challenge in Exp-2, challenge with 12,500 *E. maxima* oocysts on d 14 followed by *C. perfringens* 1 × 10^9^ challenge three times on d 19, 20, and 21; VAL, 130% additional valine diet (valine/lysine = 0.98, as a calculated value) in NE-challenged conditions; ILE, 130% additional isoleucine diet (isoleucine/lysine = 0.84, as a calculated value) in NE-challenged conditions; MIX, 130% additional valine and isoleucine diet (valine/lysine = 0.98 and isoleucine/lysine = 0.84, as a calculated value) in NE-challenged conditions. ^3^ VH:CD, villus height/crypt depth ratio.

**Table 6:** Effects of valine and isoleucine supplementation in two necrotic enteritis challenge experiments on breast muscle yield and body composition using DEXA in broilers on d 21 (7 dpi).

	NC ^1^	NE Challenge				SEM (*n* = 6)	*p*-Value
		NE	VAL	ILE	MIX		
Experiment 1 ^2^							
Breast muscle weight, g	124.0	114.7	121.3	108.7	122.0	8.78	0.720
Breast muscle weight, %	14.3	14.3	15.2	14.6	14.7	0.68	0.885
Body composition							
Total tissue weight, g	876 ^a^	784 ^ab^	780 ^ab^	692 ^b^	756 ^ab^	40.3	<0.05
Lean, g	782 ^a^	716 ^ab^	697 ^ab^	630 ^b^	680 ^ab^	34.6	<0.05
Lean, %	89.3	91.3	89.6	91.1	90.1	0.89	0.341
Fat, g	94.4	68.3	82.2	61.0	75.5	9.06	0.099
Fat, %	10.7	8.7	10.4	8.9	9.9	0.89	0.341
Body mineral composition ^3^							
BMD, mg/cm^2^	141.6	131.0	133.7	125.3	130.5	5.54	0.319
BMC, g	13.3 ^a^	10.1 ^ab^	10.9 ^ab^	8.8 ^b^	10.0 ^ab^	0.86	<0.05
BMCR, %	1.52	1.29	1.39	1.27	1.32	0.087	0.211
Experiment 2							
Breast muscle weight, g	124.0	118.7	124.0	101.3	117.3	6.79	0.146
Breast muscle weight, %	14.3	15.4	15.2	14.9	15.4	0.64	0.710
Body composition							
Total tissue weight, g	876 ^a^	734 ^b^	766 ^b^	749 ^b^	751 ^b^	30.0	<0.05
Lean, g	782 ^a^	665 ^b^	705 ^ab^	680 ^ab^	682 ^ab^	25.2	<0.05
Lean, %	89.4 ^b^	90.7	92.0	91.1	90.8	0.83	0.249
Fat, g	94.4	69.2	61.2	68.2	69.3	7.79	0.053
Fat, %	10.7	9.3	8.1	8.9	9.2	0.83	0.249
Body mineral composition ^3^							
BMD, mg/cm^2^	141.6	134.8	129.0	131.4	126.6	4.86	0.248
BMC, g	13.3 ^a^	9.6 ^b^	11.0 ^ab^	9.3 ^b^	10.2 ^b^	0.74	<0.01
BMCR, %	1.52	1.31	1.45	1.23	1.36	0.095	0.250

^a,b^ Means in the same row with different superscripts are statistically different (*p*-value < 0.05). ^1^ NC, non-challenged control (leucine/lysine = 1.12; valine/lysine = 0.76; isoleucine/lysine = 0.65, as a calculated value). ^2^ NE challenge in Exp-1, challenge with 12,500 *E. maxima* oocysts on d 14 followed by *C. perfringens* 1 × 10^9^ challenge on d 18; NE challenge in Exp-2, challenge with 12,500 *E. maxima* oocysts on d 14 followed by *C. perfringens* 1 × 10^9^ challenge three times on d 19, 20, and 21; VAL, 130% additional valine diet (valine/lysine = 0.98, as a calculated value) in NE-challenged conditions; ILE, 130% additional isoleucine diet (isoleucine/lysine = 0.84, as a calculated value) in NE-challenged conditions; MIX, 130% additional valine and isoleucine diet (valine/lysine = 0.98 and isoleucine/lysine = 0.84, as a calculated value) in NE-challenged conditions. ^3^ BMD, bone mineral density; BMC, bone mineral content; BMCR, bone mineral content ratio.

**Table 7:** Effects of valine and isoleucine supplementation in two necrotic enteritis challenge experiments on jejunal gene expression levels in broilers on d 21 (7 dpi).

	NC ^1^	NE Challenge				SEM (*n* = 6)	*p*-Value
		NE	VAL	ILE	MIX		
Experiment 1 ^2^							
Relative fold change ^3^							
*LAT1* (*SLC7A5*)	1.00 ^b^	11.01 ^a^	7.42 ^a^	7.76 ^a^	6.29 ^a^	1.182	<0.001
*LAT4* (*SLC43A2*)	1.00	0.95	1.11	1.08	1.15	0.114	0.734
*SNAT2* (*SLC38A2*)	1.00 ^b^	2.47 ^ab^	1.94 ^ab^	2.37 ^ab^	3.38 ^a^	0.382	<0.01
*y^+^LAT1* (*SLC7A7*)	1.00	1.29	1.01	1.49	1.33	0.144	0.097
*y^+^LAT2* (*SLC7A6*)	1.00	1.03	1.11	1.48	1.34	0.139	0.092
*SBAT2* (*SLC6A15*)	1.00	1.40	1.50	1.54	1.87	0.310	0.422
*BAT1* (*SLC7A9*)	1.00 ^a^	0.32 ^b^	0.42 ^b^	0.41 ^b^	0.28 ^b^	0.088	<0.001
*IL1β*	1.00 ^b^	4.86 ^a^	3.22 ^ab^	3.16 ^ab^	3.81 ^ab^	0.727	<0.05
*IL1RN*	1.00 ^b^	1.99 ^a^	1.78 ^a^	1.98 ^a^	1.66 ^a^	0.140	<0.001
*IL6*	1.00 ^b^	1.99 ^ab^	2.50 ^a^	1.98 ^ab^	2.12 ^a^	0.250	<0.01
*IL10*	1.00 ^b^	16.76 ^a^	21.64 ^a^	10.65 ^a^	11.54 ^a^	2.963	<0.001
*IFN-γ*	1.00 ^b^	13.91 ^a^	15.60 ^a^	11.62 ^a^	14.12 ^a^	2.376	<0.001
*CCL4*	1.00 ^b^	20.31 ^a^	12.56 ^a^	13.80 ^a^	13.30 ^a^	3.004	<0.01
*CXCL8*	1.00 ^b^	5.35 ^a^	4.13 ^ab^	3.06 ^ab^	3.45 ^ab^	0.823	<0.05
*TLR2*	1.00 ^b^	3.03 ^a^	2.36 ^ab^	2.88 ^a^	2.99 ^a^	0.359	<0.01
*TLR4*	1.00 ^b^	2.04 ^a^	1.49 ^ab^	1.94 ^a^	1.76 ^ab^	0.220	<0.05
*NFκB1*	1.00 ^b^	1.47 ^a^	1.41 ^a^	1.42 ^a^	1.48 ^a^	0.077	<0.001
Experiment 2							
Relative fold change							
*LAT1* (*SLC7A5*)	1.00 ^b^	7.18 ^a^	7.62 ^a^	4.63 ^a^	6.31 ^a^	0.795	<0.001
*LAT4* (*SLC43A2*)	1.00	1.03	0.93	1.25	1.05	0.112	0.357
*SNAT2* (*SLC38A2*)	1.00 ^b^	2.18 ^a^	2.26 ^a^	2.01 ^a^	2.24 ^a^	0.239	<0.01
*y^+^LAT1* (*SLC7A7*)	1.00	1.43	1.31	1.54	1.98	0.225	0.054
*y^+^LAT2* (*SLC7A6*)	1.00	1.09	1.25	0.81	0.95	0.140	0.259
*SBAT2* (*SLC6A15*)	1.00	1.40	1.50	1.54	1.87	0.310	0.422
*BAT1* (*SLC7A9*)	1.00 ^a^	0.27 ^b^	0.45 ^b^	0.20 ^b^	0.33 ^b^	0.093	<0.001
*IL1β*	1.00 ^b^	3.36 ^ab^	4.43 ^a^	4.18 ^a^	3.92 ^a^	0.701	<0.05
*IL1RN*	1.00 ^b^	1.88 ^a^	1.91 ^a^	1.79 ^a^	1.82 ^a^	0.159	<0.01
*IL6*	1.00 ^b^	2.09 ^a^	2.34 ^a^	1.81 ^ab^	2.08 ^a^	0.230	<0.01
*IL10*	1.00 ^b^	10.88 ^a^	13.85 ^a^	12.96 ^a^	11.14 ^a^	1.332	<0.001
*IFN-γ*	1.00 ^b^	10.98 ^a^	10.58 ^a^	12.81 ^a^	12.44 ^a^	1.192	<0.001
*CCL4*	1.00 ^b^	13.92 ^a^	14.90 ^a^	17.47 ^a^	13.20 ^a^	3.032	<0.01
*CXCL8*	1.00 ^b^	4.01 ^a^	3.26 ^ab^	3.99 ^a^	3.97 ^a^	0.759	<0.05
*TLR2*	1.00 ^b^	2.85 ^ab^	3.90 ^a^	2.46 ^ab^	3.49 ^a^	0.582	<0.05
*TLR4*	1.00	1.94	1.97	2.45	2.06	0.362	0.102
*NFκB1*	1.00 ^b^	1.28 ^ab^	1.47 ^a^	1.22 ^ab^	1.35 ^a^	0.084	<0.01

^a,b^ Means in the same row with different superscripts are statistically different (*p*-value < 0.05). ^1^ NC, non-challenged control (leucine/lysine = 1.12; valine/lysine = 0.76; isoleucine/lysine = 0.65, as a calculated value). ^2^ NE challenge in Exp-1, challenge with 12,500 *E. maxima* oocysts on d 14 followed by *C. perfringens* 1 × 10^9^ challenge on d 18; NE challenge in Exp-2, challenge with 12,500 *E. maxima* oocysts on d 14 followed by *C. perfringens* 1 × 10^9^ challenge three times on d 19, 20, and 21; VAL, 130% additional valine diet (valine/lysine = 0.98, as a calculated value) in NE-challenged conditions; ILE, 130% additional isoleucine diet (isoleucine/lysine = 0.84, as a calculated value) in NE-challenged conditions; MIX, 130% additional valine and isoleucine diet (valine/lysine = 0.98 and isoleucine/lysine = 0.84, as a calculated value) in NE-challenged conditions. ^3^ *LAT1* (*SLC7A5*), L-type amino acid transporter 1; *LAT4* (*SLC43A2*), L-type amino acid transporter 4; *SNAT2* (*SLC38A2*), sodium-coupled neutral amino acid transporter 2; *y^+^LAT1* (*SLC7A7*), y^+^ L-type amino acid transporter 1; *y^+^LAT2* (*SLC7A6*), y^+^ L-type amino acid transporter 2; *SBAT1* (*SLC6A15*), sodium-dependent neutral amino acid transporter 1; *BAT1* (*SLC7A9*), sodium-independent amino acid transporter 1; *IL1β*, interleukin 1 beta; *IL1RN*, interleukin 1 receptor antagonist; *IFN-γ*, interferon gamma; *CCL4*, C-C motif chemokine ligand 4; *CXCL8*, C-X-C motif chemokine ligand 8; *TLR2*, toll like receptor 2; *NFκB1*, nuclear factor kappa B subunit 1.

**Table 8:** Effects of valine and isoleucine supplementation in two necrotic enteritis challenge experiments on breast muscle gene expression levels in broilers on d 21 (7 dpi).

	NC ^1^	NE Challenge				SEM (*n* = 6)	*p*-Value
		NE	VAL	ILE	MIX		
Experiment 1 ^2^							
Relative fold change ^3^							
*mTOR*	1.00	0.74	0.82	0.75	0.96	0.083	0.081
*S6K1*	1.00 ^a^	0.69 ^b^	0.80 ^ab^	0.60 ^b^	0.76 ^ab^	0.069	<0.01
*4EBP1*	1.00	1.40	1.12	1.42	0.88	0.262	0.449
*AMPKα1*	1.00	0.76	0.81	0.72	0.88	0.079	0.120
*AKT1*	1.00	0.81	0.84	0.73	0.89	0.077	0.110
*BCAT1*	1.00 ^ab^	0.69 ^b^	1.01 ^ab^	0.70 ^b^	1.09 ^a^	0.090	<0.01
*BCKDK*	1.00	1.07	0.97	1.01	1.03	0.092	0.945
*BCKDHα*	1.00	1.10	0.96	0.96	0.93	0.062	0.340
*BCKDHβ*	1.00	1.48	0.89	1.78	1.27	0.215	0.054
*PPM1K*	1.00 ^a^	0.46 ^b^	0.59 ^ab^	0.41 ^b^	0.82 ^ab^	0.101	<0.01
*LAT1* (*SLC7A5*)	1.00 ^a^	0.61 ^ab^	1.00 ^a^	0.38 ^b^	0.65 ^ab^	0.111	<0.01
*LAT4* (*SLC43A2*)	1.00	1.99	1.72	2.33	1.43	0.329	0.077
*SNAT2* (*SLC38A2*)	1.00 ^a^	0.65 ^ab^	0.72 ^ab^	0.38 ^b^	0.68 ^ab^	0.090	<0.01
Experiment 2							
Relative fold change							
*mTOR*	1.00	1.08	1.02	0.98	0.77	0.086	0.106
*S6K1*	1.00	0.73	0.84	0.86	0.75	0.067	0.054
*4EBP1*	1.00	0.96	1.40	0.99	0.99	0.173	0.279
*AMPKα1*	1.00	1.05	0.92	0.92	0.80	0.098	0.378
*AKT1*	1.00	0.98	1.02	0.81	0.84	0.069	0.115
*BCAT1*	1.00 ^a^	0.86 ^ab^	0.99 ^a^	0.84 ^ab^	0.55 ^b^	0.079	<0.01
*BCKDK*	1.00	1.06	1.29	0.97	0.98	0.128	0.375
*BCKDHα*	1.00	1.05	1.21	1.06	0.91	0.081	0.166
*BCKDHβ*	1.00	1.51	1.75	1.14	1.20	0.232	0.173
*PPM1K*	1.00 ^a^	0.47 ^b^	0.68 ^ab^	0.59 ^ab^	0.35 ^b^	0.105	<0.01
*LAT1* (*SLC7A5*)	1.00 ^a^	0.73 ^ab^	0.57 ^b^	0.64 ^b^	0.56 ^b^	0.094	<0.01
*LAT4* (*SLC43A2*)	1.00 ^b^	4.91 ^a^	2.00 ^b^	2.42 ^b^	1.84 ^b^	0.581	<0.001
*SNAT2* (*SLC38A2*)	1.00 ^a^	0.65 ^ab^	0.61 ^ab^	0.55 ^b^	0.55 ^b^	0.096	<0.05

^a,b^ Means in the same row with different superscripts are statistically different (*p*-value < 0.05). ^1^ NC, non-challenged control (leucine/lysine = 1.12; valine/lysine = 0.76; isoleucine/lysine = 0.65, as a calculated value). ^2^ NE challenge in Exp-1, challenge with 12,500 *E. maxima* oocysts on d 14 followed by *C. perfringens* 1 × 10^9^ challenge on d 18; NE challenge in Exp-2, challenge with 12,500 *E. maxima* oocysts on d 14 followed by *C. perfringens* 1 × 10^9^ challenge three times on d 19, 20, and 21; VAL, 130% additional valine diet (valine/lysine = 0.98, as a calculated value) in NE-challenged conditions; ILE, 130% additional isoleucine diet (isoleucine/lysine = 0.84, as a calculated value) in NE-challenged conditions; MIX, 130% additional valine and isoleucine diet (valine/lysine = 0.98 and isoleucine/lysine = 0.84, as a calculated value) in NE-challenged conditions. ^3^ *mTOR*, mechanistic target of rapamycin; *S6K1*, ribosomal protein S6 kinase B1; *4EBP1*, eukaryotic translation initiation factor 4E binding protein 1; *AMPKα1*, protein kinase AMP-activated catalytic subunit alpha 1; *AKT1*, AKT serine/threonine kinase 1; *BCAT1*, branched-chain amino acid transaminase 1; *BCKDK*, branched-chain α-keto acid dehydrogenase kinase; *BCKDHα*; branched-chain α-keto acid dehydrogenase E1 subunit α; *BCKDHβ*; branched-chain α-keto acid dehydrogenase E1 subunit *β*; *PPM1K*, protein phosphatase Mg^2+^/Mn^2+^ dependent 1K; *LAT1* (*SLC7A5*), L-type amino acid transporter 1; *LAT4* (*SLC43A2*), L-type amino acid transporter 4; *SNAT2* (*SLC38A2*), sodium-coupled neutral amino acid transporter 2.

**Table 9:** Effects of valine and isoleucine supplementation in two necrotic enteritis challenge experiments on phylum-level taxonomic composition of cecal microbial communities in broilers on d 21 (7 dpi).

	NC ^1^	NE Challenge				SEM (*n* = 6)	*p*-Value
		NE	VAL	ILE	MIX		
Experiment 1 ^2^							
Relative frequency, %							
*Firmicutes*	96.0	92.2	93.7	90.8	91.4	2.40	0.574
*Bacteroidota*	1.87	4.52	1.83	8.05	3.18	3.166	0.625
F:B ratio ^3^	194	78	53	54	33	66.79	0.460
*Cyanobacteria*	0.04	0.66	1.17	0.61	1.22	0.501	0.469
*Proteobacteria*	1.05	1.45	1.39	1.86	1.31	0.334	0.559
*Actinobacteriota*	0.55	0.66	0.67	0.64	0.67	0.099	0.911
*Fusobacteriota*	0.156	0.149	0.201	0.195	0.187	0.034	0.756
*Verrucomicrobiota*	0.100	0.119	0.117	0.091	0.106	0.035	0.977
*Desulfobacterota*	0.148	0.118	0.102	0.084	0.130	0.035	0.736
*Campilobacterota*	0.076	0.055	0.053	0.051	0.070	0.028	0.961
*Patescibacteria*	0.066	0.060	0.073	0.067	0.058	0.013	0.932
Experiment 2							
Relative frequency, %							
*Firmicutes*	96.0	92.6	95.6	93.6	88.8	1.94	0.097
*Bacteroidota*	1.87	4.16	2.52	3.14	9.65	1.997	0.073
F:B ratio	194	62	53	52	18	66.9	0.407
*Cyanobacteria*	0.04	0.04	0.03	0.61	1.09	0.338	0.127
*Proteobacteria*	1.05	1.97	1.00	1.88	1.34	0.487	0.492
*Actinobacteriota*	0.55	0.84	0.56	0.74	1.22	0.163	0.054
*Fusobacteriota*	0.156	0.100	0.104	0.196	0.179	0.058	0.690
*Verrucomicrobiota*	0.100	0.112	0.069	0.140	0.135	0.033	0.570
*Desulfobacterota*	0.148	0.082	0.077	0.123	0.149	0.031	0.317
*Campilobacterota*	0.076	0.017	0.013	0.028	0.033	0.016	0.060
*Patescibacteria*	0.066	0.027	0.033	0.064	0.065	0.016	0.255

^1^ NC, non-challenged control (leucine/lysine = 1.12; valine/lysine = 0.76; isoleucine/lysine = 0.65, as a calculated value). ^2^ NE challenge in Exp-1, challenge with 12,500 *E. maxima* oocysts on d 14 followed by *C. perfringens* 1 × 10^9^ challenge on d 18; NE challenge in Exp-2, challenge with 12,500 *E. maxima* oocysts on d 14 followed by *C. perfringens* 1 × 10^9^ challenge three times on d 19, 20, and 21; VAL, 130% additional valine diet (valine/lysine = 0.98, as a calculated value) in NE-challenged conditions; ILE, 130% additional isoleucine diet (isoleucine/lysine = 0.84, as a calculated value) in NE-challenged conditions; MIX, 130% additional valine and isoleucine diet (valine/lysine = 0.98 and isoleucine/lysine = 0.84, as a calculated value) in NE-challenged conditions. ^3^ *Firmicutes* to *Bacteroidota* ratio.

**Table 10:** Effects of valine and isoleucine supplementation in two necrotic enteritis challenge experiments on family-level taxonomic composition of cecal microbial communities in broilers on d 21 (7 dpi).

	NC ^1^	NE Challenge				SEM (*n* = 6)	*p*-Value
		NE	VAL	ILE	MIX		
Experiment 1 ^2^							
Relative frequency, %							
*Ruminococcaceae*	37.7	22.6	42.4	36.5	38.8	7.19	0.371
*Clostridia vadinBB60 group*	29.5	23.5	21.8	16.1	17.5	6.68	0.644
*Bacteroidaceae*	0.79	3.72	0.93	7.29	2.21	3.142	0.581
*Lachnospiraceae*	14.3	22.8	12.0	17.9	17.5	3.34	0.234
*Oscillospiraceae*	5.9	11.1	7.6	7.1	6.6	1.37	0.104
*Clostridia UCG-014*	2.46	1.26	2.02	2.49	5.37	1.463	0.362
*Erysipelotrichaceae*	0.46	2.98	0.37	0.45	0.58	1.040	0.345
*Enterobacteriaceae*	0.44	0.85	0.53	1.16	0.60	0.299	0.448
*Lactobacillaceae*	0.56	1.24	0.86	1.28	1.23	0.238	0.178
*Butyricicoccaceae*	0.84	1.99	1.09	0.87	0.54	0.392	0.128
Experiment 2							
Relative frequency, %							
*Ruminococcaceae*	37.7	26.2	41.0	28.7	29.2	8.21	0.658
*Clostridia vadinBB60 group*	29.5	29.5	27.5	26.7	29.7	7.83	0.998
*Bacteroidaceae*	0.79	3.35	1.73	2.13	8.40	2.042	0.102
*Lachnospiraceae*	14.3	21.1	13.8	18.0	13.4	4.04	0.615
*Oscillospiraceae*	5.9	5.0	6.0	7.6	5.2	1.25	0.608
*Clostridia UCG-014*	2.46	2.56	2.52	1.80	3.47	1.509	0.958
*Erysipelotrichaceae*	0.46	0.75	0.44	0.63	0.39	0.200	0.687
*Enterobacteriaceae*	0.44	1.59	0.62	1.17	0.65	0.456	0.387
*Lactobacillaceae*	0.56	5.04	0.48	0.86	1.36	1.878	0.404
*Butyricicoccaceae*	0.84	0.40	1.24	0.37	0.63	0.289	0.220

^1^ NC, non-challenged control (leucine/lysine = 1.12; valine/lysine = 0.76; isoleucine/lysine = 0.65, as a calculated value). ^2^ NE challenge in Exp-1, challenge with 12,500 *E. maxima* oocysts on d 14 followed by *C. perfringens* 1 × 10^9^ challenge on d 18; NE challenge in Exp-2, challenge with 12,500 *E. maxima* oocysts on d 14 followed by *C. perfringens* 1 × 10^9^ challenge three times on d 19, 20, and 21; VAL, 130% additional valine diet (valine/lysine = 0.98, as a calculated value) in NE-challenged conditions; ILE, 130% additional isoleucine diet (isoleucine/lysine = 0.84, as a calculated value) in NE-challenged conditions; MIX, 130% additional valine and isoleucine diet (valine/lysine = 0.98 and isoleucine/lysine = 0.84, as a calculated value) in NE-challenged conditions.

## Data Availability

The original contributions presented in this study are included in the article. Further inquiries can be directed to the corresponding authors.

## Supplementary Materials

The following supporting information can be downloaded at https://www.mdpi.com/article/10.3390/ani15182641/s1, Table S1. Primer sequences for qRT-PCR in the current study.
