# Supplementary material for: Additional Valine and Isoleucine Impact Growth Performance, Intestinal Health, and Muscle Growth in Broilers Under Necrotic Enteritis Challenges
Source: Animals (Basel). 2025 Sep 9;15(18):2641. doi: 10.3390/ani15182641 (PMC12466562; doi:10.3390/ani15182641)
Supplement: Supplementary file 1 [file animals-15-02641-s001.zip › animals-3817305-supplementary.pdf]

Table S1. Primer sequences for qRT-PCR in the current study.

Primer[1]	Primer sequences, F/R	Product size, bp	Annealing temp., °C	Exons	Accession number / Ensembl gene ID
Amino acid transporters					
LAT1 (*SLC7A5*)	F: CCCGAGAAGGACACCTACCT<br>R: ATTGGAGAAGGCGTAGAGCA	113	61	7 / 8	NM_001030579.3<br>ENSGALG00010010461
LAT4 (*SLC43A2*)	F: CGTTCCACCTTCATTGCTCT<br>R: CTTGCCCAGACAAGCAGAAT	122	60	6 / 7	XM_415803.8<br>ENSGALG00010029341
SNAT2 (*SLC38A2*)	F: GCCGATATGGATCCAGAAAA<br>R: CCCACAATAGCATTGCTCAG	134	59	3 / 4	NM_001305439.2<br>ENSGALG00010013977
y^+LAT1 (*SLC7A7*)	F: TATTGCTGTGGCCATGTCTT<br>R: AGCAGGGACTGGTGTGAAAC	145	59	7 / 8	XM_418326.8<br>ENSGALG00010012483
y^+LAT2 (*SLC7A6*)	F: TTGGAAGGAACCAAATCGAG<br>R: ATAGAGTGGCACAACCACGA	100	59	7 / 8	XM_046899563.1<br>ENSGALG00010022992
SBAT1 (*SLC6A15*)	F: TTCACTTCTGTGCTGGCAAC<br>R: TGGCTCAGATTTCCTGTTTTC	131	59	8 / 9	XM_015283431.4<br>ENSGALG00010011117
BAT1 (*SLC7A9*)	F: ACTGGGATTGTTCTCCTTGC<br>R: CCATCATATGCCCAGAGTCC	122	59	6 / 7	NM_001199133.2<br>ENSGALG00010024831
Inflammatory cytokines					
IL1β	F: GCATCAAGGGCTACAAGCTC<br>R: CAGGCGGTAGAAGATGAAGC	131	60	4 / 5	NM_204524.2<br>ENSGALG00010018460
IL1RN	F: AACCAGCAGTCGCTGTACCT<br>R: GTGCTTGAAGAAGCGGTTG	109	60	3 / 4	NM_001397705.1<br>ENSGALG00015017924
IL6	F: GCTACAGCACAAAGCACCTG<br>R: GACTTCAGATTGGCGAGGAG	112	60	3 / 4	NM_204628.2<br>ENSGALG00010001941
IL10	F: GCTGCGCTTCTACACAGATG<br>R: CTCCTCTTCTCGCAGGTGAA	150	60	3 / 4	NM_001004414.4<br>ENSGALG00010027138
IFN-γ	F: GGCGTGAAGAAGGTGAAAGA<br>R: TCCTTTTGAAACTCGGAGGA	133	60	3 / 4	NM_205149.2<br>ENSGALG00010011933
CCL4	F: CGGGAAGATGAAGCTCTCTG<br>R: TGTAAGTGGTGCAGCAGGTC	113	60	2 / 3	NM_204720.3<br>ENSGALG00000034478
CXCL8	F: ATGTGAAGCTGACGCCAAG<br>R: GGCCATAAGTGCCTTTACGA	131	60	2 / 3	NM_205498.2<br>ENSGALG00010005131
TLR/NFκB signaling pathway-related					
TLR2	F: ACCTTCTGCACTCTGCCATT<br>R: CTTTGCGAAAGAGGAAGACA	101	58	3 / 3	NM_001161650.3<br>ENSGALG00010014865
TLR4	F: ACTCTTGGGGTGCTGCTG<br>R: TGTCCTGTGCATCTGAAAGC	110	60	1 / 2	NM_001030693.2<br>ENSGALG00010028914
NFκB1	F: TCACCAGGAGGACAACACAA<br>R: TTTGCGGAAGGAGGTCTCTA	145	60	21 / 22	NM_001396395.1<br>ENSGALG00010005476
mTOR pathway-related					
mTOR	F: AAAGCAGCTCTTCCACCAAA<br>R: TGGCTCGTGCCAACATACTA	100	60	9 / 10	XM_417614.8<br>ENSGALG00010021746
S6K1	F: TACTGGGCAAAGGTGGCTAT<br>R: ATTCCGCTCTGCTTTTGTGT	149	59	3 / 4	NM_001030721.2<br>ENSGALG00010029283
4EBP1	F: GCCCAATTGTGGAGGAGTTA<br>R: GCACGTGCTTTAGATGTCCA	106	60	2 / 3	XM_424384.8<br>ENSGALG00010019697
AMPKα1	F: TTGGGGATACGGAGTCAGAG<br>R: ACTGGATTCTTCCGTCGAAC	128	59	7 / 8	NM_001039603.2<br>ENSGALG00010014397

AKT1	F: AGCAACCGGACGGTATTATG<br>R: AGAATGGATGCCGTGAGTTC	119	60	5 / 6	NM_205055.2<br>ENSGALG00010017364
BCAA catabolism-related					
BCAT1	F: CACGATCAGCAAGACGAATG<br>R: TAGGGGACCCACTCCTTTTC	100	60	4 / 5	XM_416424.7<br>ENSGALG00010015306
BCKDK	F: CCTGGACAAGACGTTGACCT<br>R: AGTCGACCCACTTCTCGATG	143	60	7 / 8	XM_046905061.1<br>ENSGALG00010000650
BCKDHα	F: CAAGGAGCTGGTGCTGAAG<br>R: TCCTCGCCATAGTTGGTCAT	120	60	3 / 4	XM_025144505.3<br>ENSGALG00010008284
BCKDHβ	F: CAAGTGCCTTGGATAATGCTT<br>R: TGTCTCGCAAGCCAACTGTA	105	59	2 / 3	NM_204657.2<br>ENSGALG00010002431
PPM1K	F: CGCCAGAAAGGAAGGAGGAG<br>R: CTCCTATGCTCCGCGTCATT	117	60	5 / 6	XM_420574.8<br>ENSGALG00010003531
References					
ACTB	F: GTTGACAATGGCTCCGGTAT<br>R: TCTTTCTGGCCCATACCAAC	125	60	2 / 3	NM_205518.2<br>ENSGALG00010021232
GAPDH	F: CCTCTCTGGCAAAGTCCAAG<br>R: CCGTTCTCAGCCTTGACAGT	126	60	3 / 4	NM_204305.2<br>ENSGALG00010022038

[1]*LAT1* (*SLC7A5*), L-type amino acid transporter 1; *LAT4* (*SLC43A2*), L-type amino acid transporter 4; *SNAT2* (*SLC38A2*), sodium-coupled neutral amino acid transporter 2; y^+*LAT1* (*SLC7A7*), y^+ L-type amino acid transporter 1; y^+*LAT2* (*SLC7A6*), y^+ L-type amino acid transporter 2; *SBAT1* (*SLC6A15*), sodium-dependent neutral amino acid transporter 1; *BAT1* (*SLC7A9*), sodium-independent amino acid transporter 1; *IL1β*, interleukin 1 beta; *IL1RN*, interleukin 1 receptor antagonist; *IFN-γ*, interferon gamma; *CCL4*, C-C motif chemokine ligand 4; *CXCL8*, C-X-C motif chemokine ligand 8; *TLR2*, toll like receptor 2; *NFκB1*, nuclear factor kappa B subunit 1; *mTOR*, mechanistic target of rapamycin; *S6K1*, ribosomal protein S6 kinase B1; *4EBP1*, eukaryotic translation initiation factor 4E binding protein 1; *AMPKα1*, protein kinase AMP-activated catalytic subunit alpha 1; *AKT1*, AKT serine/threonine kinase 1; *BCAT1*, branched-chain amino acid transaminase 1; *BCKDK*, branched-chain α-keto acid dehydrogenase kinase; *BCKDHα*; branched-chain α-keto acid dehydrogenase E1 subunit α; *BCKDHβ*; branched-chain α-keto acid dehydrogenase E1 subunit β; *PPM1K*, protein phosphatase, Mg^{2+}/Mn^{2+} dependent 1K; *ACTB*, beta-actin; *GAPDH*, glyceraldehyde-3-phosphate dehydrogenase.
